# An elliptic Virasoro symmetry in 6d

**DOI:** 10.1007/s11005-017-0986-3

**Published:** 2017-09-04

**Authors:** Fabrizio Nieri

**Affiliations:** Department of Physics and Astronomy, Uppsala University, Box 516, 75120 Uppsala, Sweden

**Keywords:** Elliptic Virasoro algebra, Supersymmetric gauge theories, AGT, 81T20, 81T40, 81T60, 81R10, 81R50, 17B68

## Abstract

We define an elliptic deformation of the Virasoro algebra. We conjecture that the $\mathbb {R}^4\times \mathbb {T}^2$ Nekrasov partition function reproduces the chiral blocks of this algebra. We support this proposal by showing that at special points in the moduli space the 6d Nekrasov partition function reduces to the partition function of a 4d vortex theory supported on $\mathbb {R}^2\times \mathbb {T}^2$, which is in turn captured by a free field correlator of vertex operators and screening charges of the elliptic Virasoro algebra.

## Introduction

The past few years have seen significant advances in our understanding of supersymmetric gauge theories. Such progress has been largely possible due to the developments of supersymmetric localization techniques which have allowed many exact results to be obtained. One of these is the discovery by Alday, Gaiotto and Tachikawa (AGT) [1] that certain BPS observables of class $\mathcal {S}$ theories [2] of $A_1$ type can be computed in Liouville CFT, or Toda for higher rank [3]. In particular, the AGT correspondence identifies the $\mathbb {R}^4$ Nekrasov instanton partition function ($\mathcal {Z}^{\mathbb {R}^4}_{\mathrm{inst}}$) [4, 5] with the chiral blocks [6] of the Virasoro algebra (or $W_{M}$ algebra for $A_{M-1}$ theories)

$$\begin{aligned} \mathcal {Z}^{\mathbb {R}^4}_{\mathrm{inst}}\simeq { \langle \gamma _\infty |} \prod _{i=1}^N V_{\gamma _i}(x_i){ |\gamma _0\rangle }_{\mathrm{Vir}}. \end{aligned}$$

The AGT relation is a powerful tool to get further insights into the gauge dynamics as certain aspects can be efficiently addressed in the 2d CFT side, for example the study of defect operators [7–14] (for a recent review we refer to [15–17]).

It seems to be quite important to understand whether AGT-like relations exist in other dimensions as well, in particular in 6d where much of the 4d physics finds its natural origin. One of the main motivations behind this work was indeed to explore the possibility of studying 6d theories through AGT inspired methods, a topic which was previously addressed also in [18, 19]. Our results can be summarized in the proposal

$$\begin{aligned} \mathcal {Z}^{\mathbb {R}^4\times \mathbb {T}^2}_{\mathrm{inst}}\simeq { \langle \gamma _\infty |} \prod _{i=1}^N V_{\gamma _i}(x_i){ |\gamma _0\rangle }_{e\text {-Vir}}, \end{aligned}$$

where the l.h.s. captures the supersymmetric partition function of a 6d (1, 0) theory on a torus which can be engineered in M-theory by two M5-branes probing a transverse $A_{N-1}$ singularity [20], while the r.h.s. represents the chiral blocks of an elliptically deformed Virasoro algebra, which we define in this paper.

**Table 1:** The AGT relation in various dimensions

$\mathcal {Z}_{\mathrm{inst}}$ on	Chiral blocks of
$\mathbb {R}^4$	Virasoro
$\mathbb {R}^4\times S^1$	*q*-Virasoro
$\mathbb {R}^4\times \mathbb {T}^2$	*e*-Virasoro

This result can be read as the natural 1-parameter deformation (Table 1) of the 5d AGT relation [21], which we briefly recall here to pave the way for our analysis in this work. The $\mathbb {R}^4$ Nekrasov partition function and the Virasoro algebra have both a natural trigonometric deformation. The deformation of the former corresponds to the $\mathbb {R}^4\times S^1$ Nekrasov partition function [4, 5], while the deformation of the latter corresponds to the *q*-Virasoro algebra (*q*-$W_M$ algebra) of [22–24]. The identification of the two deformations predicts the 5d AGT correspondence

$$\begin{aligned} \mathcal {Z}^{\mathbb {R}^4\times S^1}_{\mathrm{inst}}\simeq { \langle \gamma _\infty |} \prod _{i=1}^N V_{\gamma _i}(x_i){ |\gamma _0\rangle }_{q\text {-Vir}}. \end{aligned}$$

Evidences supporting this idea were extensively discussed, for example, in [25–27], and more recently in [28, 29]. A complete 5d AGT relation beyond the chiral level was also proposed in [30, 31], where the $S^5$ [32–39] and $S^4\times S^1$ [40–42] partition functions of the 5d lift of class $\mathcal {S}$ theories [43] of $A_1$ type were shown to be described by correlators in two distinct QFTs with *q*-Virasoro symmetry, and hence called *q*-CFTs (see also [44–46] for an analysis of the higher rank case).

Another neat argument in favor of the 5d AGT correspondence, which we also adopt in this paper for the 6d analysis, was given in [47] (see also the review [48]). It was shown that the $\mathbb {R}^4\times S^1$ Nekrasov instanton partition function of the *U*(*N*) theory with *N* fundamental and anti-fundamental flavors reproduces, upon suitable specializations $a=a_*(r)$ of the Coulomb branch moduli, the $(N+2)$-point chiral blocks of the *q*-Virasoro algebra in the Dotsenko–Fateev free field integral representation [25, 49]

$$\begin{aligned} \mathcal {Z}^{\mathbb {R}^4\times S^1}_{\mathrm{inst}}\Big |_{a=a_*(r)}\simeq \oint \!\!\mathrm{d}z\; { \langle \gamma _{\infty }|}\prod _{i=1}^N V_{\gamma _i}(x_i)\prod _{j=1}^r S(z_j){ |\gamma _0\rangle }_{q\text {-Vir}}, \end{aligned}$$

where the operator *S*(*z*) denotes the screening current and the integral is computed by residues for specific choices of integration contours. The specialization of the Coulomb branch moduli corresponds to the root of the Higgs branch where vortex solutions exist and the dynamics can be effectively described by a 1/2 BPS codimension 2 theory on $\mathbb {R}^2_{\epsilon }\times S^1$. Partition functions of 3d $\mathcal {N}=2$ gauge theories compactified on $\mathbb {R}^2_{\epsilon }\times S^1$ can be computed by means of the 3d holomorphic block integrals ($\mathcal {B}^{\mathrm{3d}}$) introduced in [50] (see also [51] for previous work on 3d block factorization and [52] for a derivation of block integrals through localization)

$$\begin{aligned} \mathcal {B}^{\mathrm{3d}}=\oint \!\!\frac{\mathrm{d}z}{2\pi \mathrm{i}z}\Upsilon ^{\mathrm{3d}}(z), \end{aligned}$$

where the integral kernel $\Upsilon ^{\mathrm{3d}}(z)$ is a meromorphic function determined by the specific theory and the integration is over a basis of middle dimensional cycles in $(\mathbb {C}^\times )^{|G|}, G$ being the gauge group. It was pointed out in [47] that the free field integral representation of *q*-Virasoro chiral blocks manifestly matches the 3d block integrals of the *U*(*r*) theory with *N* fundamental and anti-fundamental flavors, 1 adjoint and Fayet–Iliopoulos term. Combining all these observations, one gets the identifications

$$\begin{aligned} \mathcal {Z}^{\mathbb {R}^4\times S^1}_{\mathrm{inst}}\Big |_{a=a_*(r)}\simeq \oint \!\!\mathrm{d}z\; { \langle \gamma _{\infty }|}\prod _{i=1}^N V_{\gamma _i}(x_i)\prod _{j=1}^r S(z_j){ |\gamma _0\rangle }_{q\text {-Vir}}\simeq \mathcal {B}^{\mathrm{3d}}[U(r)]. \end{aligned}$$

In particular, the two parameters *q*, *t* of the *q*-Virasoro algebra are identified with the $\epsilon _{1,2}$ parameters of the 5d $\Omega$-background $\mathbb {R}^4_{\epsilon _1,\epsilon _2}\times S^1$, and with the angular momentum fugacity $\epsilon$ and adjoint real mass in the 3d theory. The choice $a=a_*(r)$ determines the rank *r* of the 3d gauge group and the number of screening currents, which must be in turn distributed among the *N* flavors and insertion points according to a choice of partition $r=\sum _{a=1}^N r_a$. This choice corresponds to an integration contour and provides additional discrete variables (filling fractions [53–55]) entering the allowed values of the internal momenta in the correlator. This kind of “triality” can be extended to quiver gauge theories and *q*-$W_M$ correlators [56] (the generalization to *DE* root systems and applications to little string theories [57–59] can be found in [60]).

The natural lift of the 3d setup is provided by 4d $\mathcal {N}=1$ gauge theories compactified on $\mathbb {R}^2_{\epsilon }\times \mathbb {T}^2$. Partition functions on this background can be computed through the 4d holomorphic block integrals ($\mathcal {B}^{\mathrm{4d}}$) introduced in [61]. These objects can be thought of as a 1-parameter deformation of 3d block integrals, in the sense that

$$\begin{aligned} \mathcal {B}^{\mathrm{4d}}=\oint \!\!\frac{\mathrm{d}z}{2\pi \mathrm{i}z}\Upsilon ^{\mathrm{4d}}(z) ~~\xrightarrow {\text {3d limit}}~~\mathcal {B}^{\mathrm{3d}}=\oint \!\!\frac{\mathrm{d}z}{2\pi \mathrm{i}z}\Upsilon ^{\mathrm{3d}}(z). \end{aligned}$$

Due to the algebraic interpretation of 3d holomorphic blocks with adjoint matter as *q*-Virasoro chiral blocks in free field representation, we are naturally led to ask whether 4d holomorphic blocks with adjoint matter can be similarly interpreted as chiral blocks of an elliptic deformation of the Virasoro algebra. A central result of the current paper is that we can give an affirmative answer to that question, namely

$$\begin{aligned} \mathcal {B}^{\mathrm{4d}}[U(r)]\simeq \oint \!\!\mathrm{d}z\; { \langle \gamma _{\infty }|}\prod _{i=1}^N V_{\gamma _i}(x_i)\prod _{j=1}^r S(z_j){ |\gamma _0\rangle }_{e\text {-Vir}}. \end{aligned}$$

In turn, we are also able to match the evaluation of the 4d block integral with the topological string partition function on the Calabi–Yau geometry obtained by gluing two periodic strips [62–64], for special values of the Kähler moduli. The latter captures the M-theory partition function of two M5-branes extending in $\mathbb {R}^4\times \mathbb {T}^2$ and probing a transverse $A_{N-1}$ singularity, and hence we can also argue that the 4d gauge theories under examination describe vortices of 6d (1, 0) theories so engineered. This chain of results, together with large *r* duality [65–68], strongly supports a 6d version of the AGT correspondence (discussed also in [69] from the M-theory perspective) by identifying generic chiral blocks of the elliptic Virasoro algebra with $\mathbb {R}^4\times \mathbb {T}^2$ Nekrasov instanton partition functions. Lately, 6d (1, 0) theories have attracted much attention [70–81]. We hope that the methods developed in this work will be useful to get further insights into the elusive 6d physics.

The rest of this paper is organized as follows. In Sect. 2, we define an elliptic deformation of the Virasoro algebra and compute free field correlators. In Sect. 3, we briefly review the 4d block integral formalism and we consider its application to the *U*(*r*) theory with adjoint matter, manifestly matching free field correlators of the elliptic Virasoro algebra. In Sect. 4, we compute the $\mathbb {R}^4\times \mathbb {T}^2$ Nekrasov instanton partition function by using the topological vertex on the periodic strip, and we show that at specific points it coincides with the elliptic vortex partition function of the 4d theory. In Sect. 5, we discuss our results further as well as interesting directions for future research. Other and more technical aspects of this work are discussed in several appendices.

## Elliptic Virasoro Algebra

In this section we define a 1-parameter deformation of the Deformed Virasoro Algebra (DVA or *q*-Virasoro) of [22], which we call the Elliptic Virasoro Algebra (EVA or *e*-Virasoro). We give a free field representation of the EVA and find its screening currents. We then compute free field correlators of suitably defined vertex operators and screening charges. The special functions used in this section are collected in Appendix 1, while part of our notation is set in Appendix 2.

### Defining relation

We define the EVA to an be associative algebra generated by the coefficients of the current $T(z)=\sum _{n\in \mathbb {Z}}T_n z^{-n}$, with the defining relations encoded by

2.1 $$\begin{aligned}&f\left( \frac{w}{z}\right) T(z)T(w)-T(w)T(z)f\left( \frac{z}{w}\right) \nonumber \\&\quad =-\frac{\Theta (q;q')\Theta (t^{-1};q')}{(q';q')_\infty ^2\Theta (p;q')}\left( \delta \left( p\frac{w}{z}\right) -\delta \left( p^{-1}\frac{w}{z}\right) \right) , \end{aligned}$$

where the coefficients of the structure function $f(x)=\sum _{\ell \in \mathbb {Z}}f_\ell x^\ell$ are defined by the series expansion of

2.2 $$\begin{aligned} f(x)=\frac{\Gamma (x;p^2,q')\Gamma (p^2q^{-1}x;p^2,q')\Gamma (p q x;p^2,q')}{\Gamma (p^2x;p^2,q')\Gamma (p q^{-1}x;p^2,q')\Gamma (q x;p^2,q')}, \end{aligned}$$

with the elliptic Gamma function defined in (6.9) in the region $|p^2|,|q'|<1$, while $\delta (x)=\sum _{n\in \mathbb {Z}}x^n$ is the multiplicative $\delta$ function, i.e., $\delta (x)\phi (x)=\delta (x)\phi (1)$ for any Laurent series $\phi (x)$. The parameters $q,t,q'$ are complex, $p=qt^{-1}$, and for later convenience we also define $\beta \in \mathbb {C}$ such that $t=q^\beta$.

#### Remark

The associativity of the algebra is equivalent to the requirement [82, 83]

2.3 $$\begin{aligned} f(x)f(x p^{-1})-f(x^{-1})f(p x^{-1})= \kappa ( \delta (x p^{-1})-\delta (x)), \end{aligned}$$

for some constant coefficient $\kappa$, arising from the Yang–Baxter equation for *T*(*z*). The validity of (2.3) can be explicitly verified by using (2.13), (2.14), (2.15).

The choice of the structure function is motivated by the property above, the construction given in Appendix 5, and by the fact that in the limit $q'\rightarrow 0$, (2.1) manifestly reduces to the defining relation of the DVA by using

2.4 $$\begin{aligned} \Gamma (x;p^2,0)=\frac{1}{(x;p^2)_\infty },\quad \Theta (x;0)=1-x. \end{aligned}$$

This trigonometric limit can be verified at various stages of our construction below.

#### Remark

By comparing the coefficients of $z^{-n}w^{-m}$, the defining relation (2.1) is equivalent to the quadratic relation

2.5 $$\begin{aligned} \sum _{\ell \in \mathbb {Z}}f_\ell (T_{n-\ell }T_{m+\ell }-T_{m-\ell }T_{n+\ell })=-\frac{\Theta (q;q')\Theta (t^{-1};q')}{(q';q')_\infty ^2\Theta (p;q')}(p^n-p^{-n})\delta _{n+m,0}. \end{aligned}$$

### Free field representation

In order to find a free field representation of the EVA, we introduce two commuting families of quantum bosonic oscillators $\{\alpha _n,\beta _n,n\in \mathbb {Z}\backslash \{0\}\}$. They satisfy the commutation relations (we display the non-trivial relations only)

2.6 $$\begin{aligned} \begin{aligned} {[}\alpha _n,\alpha _m]&=\frac{1}{n}(1-q'^{|n|})\left( q^{\frac{n}{2}}-q^{-\frac{n}{2}}\right) \left( t^{\frac{n}{2}}-t^{-\frac{n}{2}}\right) \left( p^{\frac{n}{2}}+p^{-\frac{n}{2}}\right) \delta _{m+n,0},\\ {[}\beta _n,\beta _m]&=\frac{q'^{|n|}}{n}(1-q'^{|n|})\left( q^{\frac{n}{2}}-q^{-\frac{n}{2}}\right) \left( t^{\frac{n}{2}}-t^{-\frac{n}{2}}\right) \left( p^{\frac{n}{2}}+p^{-\frac{n}{2}}\right) \delta _{m+n,0}. \end{aligned} \end{aligned}$$

We also introduce zero mode operators $\mathsf{P},\mathsf{Q}$ commuting with all the oscillators and normalized according to

2.7 $$\begin{aligned}{}[\mathsf{P},\mathsf{Q}]=2. \end{aligned}$$

We then define the currents

2.8 $$\begin{aligned} \Lambda _{\sigma }(z)\!=\; :\mathrm{e}^{\sigma \sum _{n\ne 0}\frac{z^{-n}}{(1\!+p^{-\sigma n})(1-q'^{|n|})}\alpha _n}\mathrm{e}^{\sigma \sum _{n\ne 0}\frac{z^{n}}{(1+p^{\sigma n})(1-q'^{|n|})}\beta _n}: q^{\sigma \frac{\sqrt{\beta }}{2}\mathsf{P}}p^{\sigma \frac{1}{2}},\quad \sigma \in \{+,-\}. \end{aligned}$$

The symbol  :   :  denotes normal ordering, i.e., all the positive oscillators are placed to the right of the negative ones, and $\mathsf{P}$ to the right of the $\mathsf{Q}$. Using the commutation relations (2.6), the definition (6.9) of the elliptic Gamma function and the free boson tools summarized in Appendix 2, we can verify that the current

2.9 $$\begin{aligned} \mathsf{T}(z)=\Lambda _{+}(z)+\Lambda _{-}(z)=\sum _{n\in \mathbb {Z}}\mathsf{T}_n z^{-n}~ \end{aligned}$$

satisfies the defining relation (2.1) of the EVA. The explicit verification of this claim is straightforward but lengthy, and hence presented in Appendix 3. The key relations to be used are

2.10 $$\begin{aligned} \Lambda _\sigma (z)\Lambda _\rho (w)=\; :\Lambda _\sigma (z)\Lambda _\rho (w):f_{\sigma ,\rho }(w z^{-1})^{-1},\quad (\sigma ,\rho )\in \{\pm ,\pm \}, \end{aligned}$$

where

2.11 $$\begin{aligned} f_{\sigma ,\rho }(x)=\left( \frac{\Gamma \left( p^{\frac{\sigma \cdot 1-\rho \cdot 1}{2}}x;p^2,q'\right) \Gamma \left( p^{\frac{\sigma \cdot 1-\rho \cdot 1}{2}}p^2q^{-1}x;p^2,q'\right) \Gamma \left( p^{\frac{\sigma \cdot 1-\rho \cdot 1}{2}}p q x;p^2,q'\right) }{\Gamma \left( p^{\frac{\sigma \cdot 1-\rho \cdot 1}{2}}p^2x;p^2,q'\right) \Gamma \left( p^{\frac{\sigma \cdot 1-\rho \cdot 1}{2}}p q^{-1}x;p^2,q'\right) \Gamma \left( p^{\frac{\sigma \cdot 1-\rho \cdot 1}{2}}q x;p^2,q'\right) }\right) ^{\sigma \rho \;\cdot 1},\nonumber \\ \end{aligned}$$

and2.12One also needs

2.13 $$\begin{aligned} f(x)=f_{+,+}(x)=f_{-,-}(x)=f_{+,-}(x)\gamma \left( p^{\frac{1}{2}}x\right) =f_{-,+}(x)\gamma \left( p^{-\frac{1}{2}}x\right) , \end{aligned}$$

where

2.14 $$\begin{aligned} \gamma (x)=\frac{\Theta (p^{\frac{1}{2}}q^{-1}x;q')\Theta (p^{-\frac{1}{2}}q x;q')}{\Theta (p^{\frac{1}{2}}x;q')\Theta (p^{-\frac{1}{2}}x;q')}, \end{aligned}$$

as well as the equality

2.15 $$\begin{aligned} \gamma (x)-\gamma (x^{-1})=-\frac{\Theta (q;q')\Theta (t^{-1};q')}{(q';q')_\infty ^2\Theta (p;q')}\left( \delta (p^{\frac{1}{2}}x)-\delta (p^{-\frac{1}{2}}x)\right) , \end{aligned}$$

which follows from the representation (8.14) of the $\delta$ function. More details along with technical comments are given in Appendix 3.

#### Remark

Using $(M-1)$-dimensional oscillators $\vec \alpha _n, \vec \beta _n$ associated with the $A_{M-1}$ root system, it is possible to extend our construction to define an elliptic version of the $W_{M}$ algebra, along the lines of [23, 24, 84].

### Screening currents

The screening current $\mathsf{S}(z)$ of the EVA in the free field representation (2.9) is defined by the relation

2.16 $$\begin{aligned}{}[\mathsf{T}_n,\mathsf{S}(w)]=\frac{\mathrm{d}}{\mathrm{d}_q w}\mathsf{A}_n(w)=\frac{\mathsf{A}_n(q^{\frac{1}{2}}w)-\mathsf{A}_n\left( q^{-\frac{1}{2}}w\right) }{w\left( q^{\frac{1}{2}}-q^{-\frac{1}{2}}\right) } \end{aligned}$$

for some operator $\mathsf{A}_n(w)$, so that the EVA generators and the screening charge $\oint \!\! \mathrm{d}w\;\mathsf{S}(w)$ commute for a suitable *q*-invariant integration contour (e.g., around the origin). With the definition

2.17 $$\begin{aligned} \mathsf{S}(z)=\, :\mathrm{e}^{-\sum _{n\ne 0}\frac{z^{-n}}{\left( q^{n/2}-q^{-n/2}\right) (1-q'^{|n|})}\alpha _n}\mathrm{e}^{\sum _{n\ne 0}\frac{z^{n}}{\left( q^{n/2}-q^{-n/2}\right) (1-q'^{|n|})}\beta _n}:\mathrm{e}^{\sqrt{\beta }\mathsf{Q}}z^{\sqrt{\beta }\mathsf{P}}, \end{aligned}$$

we can verify that (2.16) is satisfied. Since this particular choice of screening current will be crucial for actual computations, in Appendix 4 we explicitly show how the claim (2.16) can be verified.

#### Remark

Another screening current can also be defined through the map $q\rightarrow t, t\rightarrow q, \alpha _n\rightarrow -\alpha _n, \beta _n\rightarrow -\beta _n, \sqrt{\beta }\rightarrow -1/\sqrt{\beta }$, but we do not need it for our purposes.

The product of several screening currents can be written as

2.18 $$\begin{aligned} \prod _{i=1}^r\mathsf{S}(z_i)= & {} \; :\prod _{i=1}^r\mathsf{S}(z_i): \times \prod _{1\le i\ne j\le r}\frac{\Gamma (t z_j z_i^{-1};q,q')}{\Gamma (z_j z_i^{-1};q,q')}\prod _{1\le i<j\le r}\frac{\Theta (t z_i z_j^{-1};q)}{\Theta (z_i z_j^{-1};q)}\nonumber \\&\times \prod _{i=1}^{r}z_i^{2\beta (r-i)} . \end{aligned}$$

The last factor arises from the normal ordering of the zero modes

2.19 $$\begin{aligned} \prod _{i}\left( \mathrm{e}^{\sqrt{\beta }\mathsf{Q}}z_i^{\sqrt{\beta }\mathsf{P}}\right) = \prod _{i}\mathrm{e}^{\sqrt{\beta }\mathsf{Q}}\prod _{i}z_i^{\sqrt{\beta }\mathsf{P}}\times \prod _{i<j}z_i^{2 \beta }, \end{aligned}$$

and it can also be rewritten as

2.20 $$\begin{aligned} \prod _{i<j}z_i^{2 \beta }=\prod _{i=1}^r z_i^{2\beta (r-i)}=\prod _{i=1}^r\frac{z_i^{\sqrt{\beta }(\sqrt{\beta }r-Q)}}{z_i}\times \prod _{1\le i<j\le r}\left( \frac{z_i}{z_j}\right) ^\beta , \end{aligned}$$

where we defined

2.21 $$\begin{aligned} Q=\sqrt{\beta }-\frac{1}{\sqrt{\beta }}. \end{aligned}$$

The last factor in the r.h.s. of (2.20) can be put with the $\Theta$s in (2.18) to form the *q*-constant^1^

2.22 $$\begin{aligned} c_\beta (z;q)=\prod _{1\le i<j\le r}\left( \frac{z_i}{z_j}\right) ^\beta \frac{\Theta (t z_i z_j^{-1};q)}{\Theta (z_i z_j^{-1};q)}, \end{aligned}$$

while we can use the product of the $\Gamma$s in (2.18) to define an elliptic Vandermonde-like determinant

2.23 $$\begin{aligned} \Delta _E(z)=\prod _{1\le i\ne j\le r}\frac{\Gamma (t z_i z_j^{-1};q,q')}{\Gamma (z_i z_j^{-1};q,q')}. \end{aligned}$$

When considering free field correlators with integrated screening currents, this object will provide the integration measure. In fact, we can simply forget about the *q*-constant (2.22) because of the integration contour that we will prescribe (see discussion in Sect. 3).

### Vertex operators

Let us define the following vertex operator built out of the bosonic oscillators and zero modes

2.24 $$\begin{aligned} \mathsf{V}_u(x)=\; :\mathrm{e}^{\sum _{n\ne 0}\frac{[u]_n x^{-n}}{\left( q^{n/2}-q^{-n/2}\right) (1-q'^{|n|})}\alpha _n} \mathrm{e}^{-\sum _{n\ne 0}\frac{[u]_n x^{n}}{\left( q^{n/2}-q^{-n/2}\right) (1-q'^{|n|})}\beta _n}:\mathrm{e}^{-\frac{\gamma }{2}\sqrt{\beta }\mathsf{Q}}x^{-\frac{\gamma }{2}\sqrt{\beta }\mathsf{P}}, \end{aligned}$$

where

2.25 $$\begin{aligned}{}[u]_n=\frac{u^{\frac{n}{2}}-u^{-\frac{n}{2}}}{(t^{\frac{n}{2}}-t^{-\frac{n}{2}})(p^{\frac{n}{2}}+p^{-\frac{n}{2}})},\quad u=t^\gamma . \end{aligned}$$

The momentum $\gamma$ (or equivalently *u*) is a free parameter labeling the vertex operator. The “OPE” between this vertex operator and the screening current can be written as

2.26 $$\begin{aligned} \mathsf{V}_u(x)\mathsf{S}(z) =\; :\mathsf{V}_u(x)\mathsf{S}(z):\times \frac{\Gamma (q^{\frac{1}{2}}u^{-\frac{1}{2}}z x^{-1};q,q')}{\Gamma (q^{\frac{1}{2}}u^{\frac{1}{2}}z x^{-1};q,q')}\times x^{-\beta \gamma }, \end{aligned}$$

where the last factor arises from the normal ordering of the zero modes

2.27 $$\begin{aligned} x^{-\frac{\gamma }{2}\sqrt{\beta }\mathsf{P}}\mathrm{e}^{\sqrt{\beta }\mathsf{Q}}= \mathrm{e}^{\sqrt{\beta }\mathsf{Q}} x^{-\frac{\gamma }{2}\sqrt{\beta }\mathsf{P}}\times x^{-\beta \gamma }. \end{aligned}$$

In the following, we do not need the explicit form of the “OPE” between vertex operators alone.

### Correlators

Let us start by defining the zero momentum Fock space $\mathcal {F}_0$. It is the left module over the oscillator algebra (2.6) generated by the vacuum ${ |0\rangle }$ defined by

2.28 $$\begin{aligned} \alpha _{n}{ |0\rangle }=\beta _{n}{ |0\rangle }=0,\quad n\in \mathbb {Z}_{>0}, \end{aligned}$$

namely

2.29 $$\begin{aligned} \mathcal {F}_0=\mathrm{Span}\Big \{\alpha _{-\mu }\beta _{-\nu }{ |0\rangle },\mu ,\nu \in \mathcal {P}\Big \}, \end{aligned}$$

where $\mathcal {P}$ is the set of partitions, and for length $\ell (\mu ), \ell (\nu )$ partitions we defined $\alpha _{-\mu }=\alpha _{-\mu _1}\;\cdots \;\alpha _{-\mu _{\ell (\mu )}}, \beta _{-\nu }=\beta _{-\nu _1}\;\cdots \;\beta _{-\nu _{\ell (\nu )}}$. Let us also define the dual (right) Fock module

2.30 $$\begin{aligned} \mathcal {F}_0^*=\mathrm{Span}\Big \{{ \langle 0|}\alpha _{\mu }\beta _{\nu },\mu ,\nu \in \mathcal {P}\Big \}, \end{aligned}$$

generated by the dual vacuum ${ \langle 0|}$ defined by ${ \langle 0|}\alpha _{-n}\beta _{-m}=0, n,m\in \mathbb {Z}_{>0}$.^2^ Acting with the exponential of the zero mode operator $\mathsf{Q}$ on the neutral vacua, we can define charged Fock vacua generating the charged Fock modules $\mathcal {F}_\gamma , \mathcal {F}^*_\gamma$

2.31 $$\begin{aligned} { |\gamma \rangle }=\mathrm{e}^{\frac{\gamma }{2} {\mathsf{Q}}}{ |0\rangle },\quad { \langle \gamma |}={ \langle 0|}\mathrm{e}^{-\frac{\gamma }{2}{\mathsf{Q}}},\quad \gamma \in \mathbb {C}. \end{aligned}$$

The charged vacua are eigenstates of the momentum operator $\mathsf{P}$

2.32 $$\begin{aligned} \mathsf{P}{ |\gamma \rangle }=\gamma { |\gamma \rangle },\quad { \langle \gamma |}\mathsf{P}=\gamma { \langle \gamma |} , \end{aligned}$$

and we define the following pairing between the left and right charged Fock modules

2.33 $$\begin{aligned} { \left\langle \gamma |\gamma ' \right\rangle }=\delta _{\gamma ,\gamma '}. \end{aligned}$$

We are now ready to compute correlators of *N* vertex operators and *r* integrated screening currents between external Fock states. We start by using the manipulations of the previous subsection to write

2.34 $$\begin{aligned}&\prod _{i=1}^N\mathsf{V}_{u_i}(x_i)\prod _{i=1}^r\mathsf{S}(z_i)=\; :\prod _{i=1}^N\mathsf{V}_{u_i}(x_i)\prod _{i=1}^r\mathsf{S}(z_i):\times \mathrm{``OPE''}\times \prod _{j=1}^N x_j^{-\beta r\gamma _j}\nonumber \\&\quad \times c_\beta (z;q)\times \Delta _E(z)\prod _{i=1}^r\frac{z_i^{\sqrt{\beta }(\sqrt{\beta }r-Q)}}{z_i}\prod _{i=1}^r\prod _{j=1}^N \frac{\Gamma (q^{\frac{1}{2}}u_j^{-\frac{1}{2}}z_i x_j^{-1};q,q')}{\Gamma (q^{\frac{1}{2}}u_j^{\frac{1}{2}}z_i x_j^{-1};q,q')},\quad \end{aligned}$$

where we set $u_j=t^{\gamma _j}$. The $\mathrm{``OPE''}$ prefactor denotes all the normal ordering terms arising from vertex operators alone, which are not important for the present analysis and hence will be neglected in the following. Sandwiching (2.34) between two Fock states ${ |\gamma _0\rangle }$ and ${ \langle \gamma _\infty |}$ we get (up to constant, i.e., $z_i$-independent factors)

2.35 $$\begin{aligned}&{ \langle \gamma _\infty |}\prod _{i=1}^N \mathsf{V}_{u_i}(x_i)\prod _{i=1}^r\mathsf{S}(z_i){ |\gamma _0\rangle }\propto { \left\langle \gamma _\infty |\gamma _0+\sqrt{\beta }(2r-\sum _i\gamma _i) \right\rangle }\nonumber \\&\quad \times c_\beta (z;q)\times \Delta _E(z)\prod _{i=1}^r\frac{z_i^{\sqrt{\beta }(\gamma _0+\sqrt{\beta }r-Q)}}{z_i} \prod _{i=1}^r\prod _{j=1}^N\frac{\Gamma (q^{\frac{1}{2}}u_j^{-\frac{1}{2}}z_i x_j^{-1};q,q')}{\Gamma (q^{\frac{1}{2}}u_j^{\frac{1}{2}}z_i x_j^{-1};q,q')},\nonumber \\ \end{aligned}$$

which is nonzero provided the neutrality condition $\sqrt{\beta }(2r-\sum _{i=1}^N \gamma _i)+\gamma _0-\gamma _\infty =0$ holds. In this case, the correlator integrated over the positions of the screening currents reads

2.36 $$\begin{aligned}&G^{(N)}_{\gamma _\infty ,\gamma _0}=\oint \prod _{i=1}^r\frac{\mathrm{d}z_i}{2\pi \mathrm{i}}{ \langle \gamma _\infty |}\prod _{i=1}^N \mathsf{V}_{u_i}(x_i)\prod _{i=1}^r\mathsf{S}(z_i){ |\gamma _0\rangle }\nonumber \\&\quad \propto \oint \prod _{i=1}^r\frac{\mathrm{d}z_i}{2\pi \mathrm{i}z_i}\;c_\beta (z;q)\;\Delta _E(z)\prod _{i=1}^r z_i^{\sqrt{\beta }(\gamma _0+\!\sqrt{\beta }r-Q)}\prod _{i=1}^r\prod _{j=1}^N \frac{\Gamma (q^{\frac{1}{2}}u_j^{\!-\frac{1}{2}}z_i x_j^{-1};q,q')}{\Gamma (q^{\frac{1}{2}}u_j^{\frac{1}{2}}z_i x_j^{-1};q,q')}, \end{aligned}$$

where we left the integration contour momentarily unspecified. For later purposes, it is useful to reorder the integration variables $z_i\rightarrow z_{r-i+1}$, perform the change $z_i\rightarrow z_i^{-1}$, and set $q^{1/2}u_j^{-1/2}x_j^{-1}=y_j$. The combination of these transformations acts as the identity on $\Delta _E(z)$ and $c_\beta (z;q)$, and then, we have

2.37 $$\begin{aligned} G^{(N)}_{\gamma _\infty ,\gamma _0}\propto \oint \prod _{i=1}^r\frac{\mathrm{d}z_i}{2\pi \mathrm{i}z_i}\;c_\beta (z;q)\;\Delta _E(z)\prod _{i=1}^r z_i^{\sqrt{\beta }(Q-\gamma _0\!-\!\sqrt{\beta }r)}\prod _{i=1}^r\prod _{j=1}^N\frac{\Gamma (y_j z_i^{-1};q,q')}{\Gamma (u_j y_j z_i^{-1};q,q')}. \end{aligned}$$

This integral looks like an elliptic deformation of the Dotsenko–Fateev representation of the chiral blocks of the DVA.^3^ In fact, in the trigonometric limit $q'\rightarrow 0$ we get

2.38 $$\begin{aligned}&\lim _{q'\rightarrow 0}G^{(N)}_{\gamma _\infty ,\gamma _0}\propto \oint \prod _{i=1}^r\frac{\mathrm{d}z_i}{2\pi \mathrm{i}z_i}\;c_\beta (z;q)\;\Delta _T(z)\prod _{i=1}^r z_i^{\sqrt{\beta }(Q-\gamma _0-\sqrt{\beta }r)}\nonumber \\&\quad \times \prod _{i=1}^r\prod _{j=1}^N \frac{(u_j y_j z_i^{-1};q)_\infty }{(y_j z_i^{-1};q)_\infty }, \end{aligned}$$

where

2.39 $$\begin{aligned} \Delta _T(z)=\prod _{1\le i\ne j\le r}\frac{(z_i z_j^{-1};q)_\infty }{(t z_i z_j^{-1};q)_\infty } \end{aligned}$$

is the trigonometric Vandermonde-like determinant appearing in the *q*-deformed $\beta$-ensemble studied, for example, in [25] in the context of the 5d AGT correspondence. In the special case $t=q^\beta , \beta \in \mathbb {Z}_{>0}$, we find

2.40 $$\begin{aligned} \Delta _E(z)=\prod _{1\le i\ne j\le r}\prod _{k=0}^{\beta -1}\Theta (q^k z_j z_i^{-1};q'), \end{aligned}$$

which in the trigonometric limit $q'\rightarrow 0$ reduces to

2.41 $$\begin{aligned} \Delta _T(z)=\prod _{1\le i\ne j\le r}\prod _{k=0}^{\beta -1}(1-q^k z_j z_i^{-1}). \end{aligned}$$

The latter represents, apart for a factor of $\prod _{i=1}^r z_i^{-\beta (r-1)}$ which can be reabsorbed into the integrand factors, the ordinary *q*-deformation [26] of the $\beta$-deformed rational Vandermonde determinant

2.42 $$\begin{aligned} \Delta _R(z)=\prod _{1\le i<j\le r}(z_i-z_j)^{2\beta }. \end{aligned}$$

## 4d holomorphic blocks

**Fig. 1:**
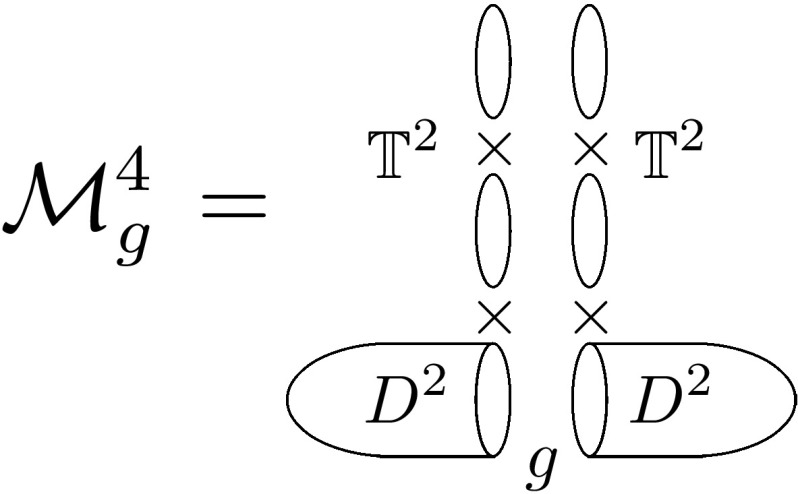
Decomposition of $\mathcal {M}^4_g$ into solid tori $D^2\times \mathbb {T}^2$

In [61] we have analyzed the structure of supersymmetric partition functions of 4d $\mathcal {N}=1$ theories with R-symmetry on compact manifolds ($\mathcal {M}^4_g$). This class of theories can be coupled to new minimal supergravity backgrounds [85], and in the rigid limit [86] 2 supercharges of opposite R-charge can be preserved. In this case $\mathcal {M}^4_g$ must be a Hermitian manifold given by a $\mathbb {T}^2$ fibration over a Riemann surface. When the base has the topology of $S^2, \mathcal {M}^4_g$ can be considered to be $S^3\times S^1, S^3/\mathbb {Z}_k\times S^1$ or $S^2\times \mathbb {T}^2$. From our viewpoint, $\mathcal {M}^4_g$ is not an elementary geometry in the sense that it admits a Heegaard-like splitting into solid tori $D^2 \times \mathbb {T}^2\simeq \mathbb {R}^2_\epsilon \times \mathbb {T}^2$ (Fig. 1)

3.1 $$\begin{aligned} \mathcal {M}^4_g\simeq (D^2\times \mathbb {T}^2)\cup _g (D^2\times \mathbb {T}^2), \end{aligned}$$

where *g* represents a certain element in $SL(3,\mathbb {Z})$ implementing the $\mathbb {T}^3$ boundary homeomorphism realizing the compact geometry and acting on the fibration moduli $\tau ,\sigma$. In this construction $\tau \propto \epsilon$ is to be identified with the disk equivariant parameter ($\Omega$-deformation) while $\sigma$ with the torus modular parameter. Eventually, one can map $\tau$ and $\sigma$ to the complex structure parameters of $\mathcal {M}^4_g$ (see, for example, the discussion in [87, 88] and references therein).

These geometric observations acquire even more importance if we recall that the compact space partition functions of the class of theories we are considering are quasi-topological objects, as they depend on the complex structure but do not depend on the Hermitian metric [85, 89]. Assuming that there are deformations of $\mathcal {M}_g^4$ into a stretched geometry which preserve the complex structure (similarly to the 3d case [90]), one expects that the associated partition function (*Z*) can be factorized according to the underlying geometric decomposition of $\mathcal {M}_g^4$

3.2 $$\begin{aligned} Z_{\mathcal {M}_g^4}=\sum _c \Big \Vert \mathcal {B}_c^{\mathrm{4d}}\Big \Vert ^2_g, \end{aligned}$$

where $\mathcal {B}_c^{\mathrm{4d}}$ is identified with the $\mathbb {R}_\epsilon ^2\times \mathbb {T}^2$ partition function of the 4d theory and *c* runs over the supersymmetric vacua of the effective 2d theory. The functions $\mathcal {B}_c^{\mathrm{4d}}$ were called 4d holomorphic blocks [61] because the *g*-pairing acts as an involution mapping a block to the conjugate block, where the *g*-action is on $\tau , \sigma$ and on a set of variables parametrizing global fugacities.

In [61] we have explicitly proved this structure in rank 1 gauge theories and argued its general validity for anomaly free theories by means of other arguments, such as the existence of a commuting set of difference operators annihilating the partition functions, Higgs branch localization [91] or the close relation to $tt^*$ geometries [92]. In fact, for a given gauge theory we have found a specific recipe to compute $\mathcal {B}_c^{\mathrm{4d}}$ through a block integral formalism, similar to that developed in [50] for the 3d case. The fundamental object of this formalism is an integral kernel $\Upsilon ^{\mathrm{4d}}(z)$ whose contour integrals produce the 4d holomorphic blocks^4^

3.3 $$\begin{aligned} \mathcal {B}_c^{\mathrm{4d}}=\oint _{\mathcal {P}_c}\frac{\mathrm{d}z}{2\pi \mathrm{i}z}\Upsilon ^{\mathrm{4d}}( z), \end{aligned}$$

where $\mathcal {P}_c$ belongs to a basis of middle dimensional integration cycles in $(\mathbb {C}^{\times })^{|G|}$ which can be determined by the specific matter content and gauge group *G*. The kernel $\Upsilon ^{\mathrm{4d}}(z)$ can be assembled using the rules derived in [61], which can be briefly summarized as follows (we refer to [61] for a full account):

- To a vector multiplet we associate a factor of
  3.4 $$\begin{aligned} \mathcal {B}_{\mathrm{vec}}^{\mathrm{4d}}( z)= \prod _{\alpha }\frac{1}{\Gamma (z_\alpha ;q_\tau ,q_\sigma )}, \end{aligned}$$
  where $\alpha$ denotes a gauge root.
- To a chiral multiplet we associate a factor of
  3.5 $$\begin{aligned} \mathcal {B}_{\mathrm{N}}^{\mathrm{4d}}( z, x)=\prod _{\rho }\Gamma (z_\rho x;q_\tau ,q_\sigma ),\quad \text { or }\quad \mathcal {B}_{\mathrm{D}}^{\mathrm{4d}}( z, x)=\prod _{\rho }\frac{1}{\Gamma (q_\tau z_\rho ^{-1} x^{-1};q_\tau ,q_\sigma )}, \end{aligned}$$
  where $\rho$ is a gauge weight while *x* is a global *U*(1) fugacity.

The parameters $q_\tau =\mathrm{e}^{2\pi \mathrm{i}\tau }$ and $q_\sigma =\mathrm{e}^{2\pi \mathrm{i}\sigma }$ can be interpreted as fugacities for rotations on the disk and translations on the torus. We should also observe that the construction of the integral kernel suffers from some ambiguity represented by $q_\tau$-constants.^5^

We can now apply the 4d block integral formalism to the higher rank example we have mentioned in the introduction, namely the *U*(*r*) theory with *N* fundamentals and anti-fundamental chirals, 1 adjoint and FI parameter ($\xi$). Using the rules summarized above, the block integral for this theory can be written as

3.6 $$\begin{aligned} \mathcal {B}^{\mathrm{4d}}= & {} \oint \prod _{i=1}^r\frac{\mathrm{d}z_i}{2\pi \mathrm{i}z_i}\Upsilon ^{\mathrm{4d}}(z),\nonumber \\ \Upsilon ^{\mathrm{4d}}(z)= & {} \prod _{1\le i\ne j\le r}\frac{\Gamma (t z_i z_j^{-1};q_\tau , q_\sigma )}{\Gamma (z_i z_j^{-1};q_\tau , q_\sigma )}\prod _{i=1}^{r}z_i^\xi \prod _{a=1}^N \frac{\Gamma (y_a z_i^{-1};q_\tau ,q_\sigma )}{\Gamma (u_a y_a z_i^{-1};q_\tau , q_\sigma )}, \end{aligned}$$

where for later convenience the global parameters have been encoded into *t*, *u*, *y*.^6^

In this parametrization the 4d block integral (3.6) is manifestly equal (up to prefactors) to the correlator (2.37) that we have introduced in the previous section

3.7 $$\begin{aligned} \mathcal {B}^{\mathrm{4d}}\propto G^{(N)}_{\gamma _\infty ,\gamma _0}, \end{aligned}$$

where the identification of parameters is as follows

3.8 $$\begin{aligned} \begin{array}{c|c|c|c|c|c|c} \text {Gauge theory}~&{}~ q_\tau ~&{}~q_\sigma ~&{}~t~&{}~u~&{}~y~&{}~\xi \\ \hline \text {EVA}~&{}~q~&{}~q'~&{}~t~&{}~u~&{}~y~&{}~Q-\gamma _0-\sqrt{\beta }r~ \end{array}. \end{aligned}$$

In particular, the gauge theory integration measure given by the adjoint and vector multiplets and the elliptic Vandermonde-like determinant (2.23) coming from “OPE” factor of the screening currents are identified. As we mentioned around (2.22), the actual measures may differ by *q*-constants, but they give the same result (up to proportionality factors) when integrating along paths enclosing the poles specified in the following (3.14).^7^ It then follows that (3.6) can be interpreted as the Dotsenko–Fateev representation of the chiral blocks of the EVA, as summarized in (3.7).

We now turn to discussing the integration contour ($\mathcal {C}$), which was left unspecified so far, and the evaluation of the block integral/correlator (3.6) by residues. We assume $|q_\tau |<1, |q_\sigma |<1, |t|<1$ and that they are generic, namely $q_\tau ^m\ne q_\sigma ^n\ne t^k$ for any $m,n,k\in \mathbb {Z}\backslash \{0\}$. We begin by studying the pole distribution of the block integrand in (3.6), focusing on the *u*-independent ones. These are associated with anti-fundamental matter in our conventions, and they determine $\mathcal {C}$ as we are going to explain. The poles coming from the numerator of the matter contribution are located at

3.9 $$\begin{aligned} z_i=y_a q_\tau ^n q_\sigma ^k,\quad n,k\in \mathbb {Z}_{\ge 0}. \end{aligned}$$

Further poles come from the numerator of the integration measure (adjoint) and are determined by the condition

3.10 $$\begin{aligned} \frac{z_i}{z_j}=t q_\tau ^n q_\sigma ^k,\quad n,k\in \mathbb {Z}_{\ge 0}. \end{aligned}$$

Importantly, there are zeros coming from the denominator of the integration measure (vector) whenever

3.11 $$\begin{aligned} \frac{z_i}{z_j}=q_\tau ^n q_\sigma ^k,\quad n,k\in \mathbb {Z}_{\ge 0}. \end{aligned}$$

The contour $\mathcal {C}$ is chosen to encircle only the poles of the form

3.12 $$\begin{aligned} z_i=y_a t ^k q_\tau ^n,\quad n,k\in \mathbb {Z}_{\ge 0}. \end{aligned}$$

As explained in detail in [61], this prescription arises by interpreting the contributions from poles containing powers of $q_\sigma$ as unphysical replicas because of the quasi-periodicity on $\mathbb {T}^2$. Moreover, this prescription works in rank 1 examples [61], and we adopt it also in the present case. In order to completely specify the integration path, we split the integration variables into *N* groups of $r_{a}$, namely

3.13 $$\begin{aligned} r=\sum _{a=1}^{N}r_a,\quad \{z_i\}=\{z_{(a,\ell )}~|~ a=1,\ldots ,N,~\ell =1,\dots ,r_a\}, \end{aligned}$$

and we assign a contour to each group. Within each group, the sequence $z_{(a,\ell )}=y_a q_\tau ^n$ connects the points $z_{(a,\ell )}=0$ and $z_{(a,\ell )}=y_a$, and the contour is taken to go around that path. In order to understand what are the contributing poles, let us focus on the *a* ^th^ group. First of all, we have a permutation symmetry among the $z_{(a,\ell )}$ which we fix by starting to integrate from the last variable of the group all the way to the first one, namely we perform the integrations in the order $z_{(a,r_a)},z_{(a,r_a-1)},\ldots ,z_{(a,1)}$. For the last variable the contributing poles are just those from the anti-fundamentals, namely $z_{(a,r_a)}=y_a q_\tau ^n$. Then, we perform the integration over the next-to-last variable $z_{(a,r_a-1)}$. The possible contributing poles arise from the anti-fundamentals at $z_{(a,r_a-1)}=y_a q_\tau ^{k}$, or from the adjoint at $z_{(a,r_a-1)}=y_a t q_\tau ^{n+k}$. The first family does not contribute because the condition $\frac{z_{(a,r_a-1)}}{z_{(a,r_a)}}=q_\tau ^\mathbb {Z}$ is satisfied and hence the vector contributes with a zero. Similarly, for the variable $z_{(a,r_a-2)}$ we find the contributing poles are those at $z_{(a,r_a-2)}=y_a t^2 q_\tau ^{n+k+j}$, and so on. The same reasoning applies to each group, and we can eventually realize that the relevant poles are labeled by an *N*-tuple of Young tableaux $Y^a$ with at most $r_a$ rows of length $Y^a_\ell$

3.14 $$\begin{aligned} z_{(a,\ell )}=z_{Y^a_\ell }=y_a t^{r_a-\ell }q_\tau ^{Y^a_\ell },\quad Y^a_\ell \ge Y^a_{\ell +1}. \end{aligned}$$

The sum of the residues of (3.6) over these poles can be evaluated by using the properties in (6.12), and we can finally write

3.15 $$\begin{aligned} \mathcal {B}^{\mathrm{4d}}_\mathcal {C}=\mathrm{Res}_{z=z_{\vec \emptyset }}\frac{\Upsilon ^{\mathrm{4d}}(z)}{z}\times \sum _{\vec Y}\frac{\Upsilon ^{\mathrm{4d}}(z)|_{z_{\vec Y}}}{\Upsilon ^{\mathrm{4d}}( z)|_{z_{\vec \emptyset }}}. \end{aligned}$$

The summands of the series reads as

3.16 $$\begin{aligned} \frac{\Upsilon ^{\mathrm{4d}}(z)|_{z_{\vec Y}}}{\Upsilon ^{\mathrm{4d}}(z)|_{z_{\vec \emptyset }}}= & {} q_\tau ^{\xi |\vec Y|}\!\!\!\!\!\!\!\prod _{(a,\ell )\ne (b,k)}\!\!\!\frac{\Theta (t y_a y_b^{-1}t^{r_a-r_b-\ell +k};q_\sigma ,q_\tau )_{Y^a_\ell -Y^b_k}}{\Theta (y_a y_b^{-1}t^{r_a-r_b-\ell +k};q_\sigma ,q_\tau )_{Y^a_\ell -Y^b_k}}\nonumber \\&\prod _{a,b,\ell }\!\frac{\Theta (y_b y_a^{-1} t^{-r_a+\ell };q_\sigma ,q_\tau )_{-Y^a_\ell }}{\Theta (u_b y_b y_a^{-1} t^{-r_a+\ell };q_\sigma ,q_\tau )_{-Y^a_\ell }}, \end{aligned}$$

where $|\vec Y|\!=\!\sum _{a=1}^{N}\!\sum _{\ell =1}^{r_a}\!Y^a_\ell$ and the $\Theta$-factorial is defined in (6.14). We see that $\mathcal {B}^{\mathrm{4d}}_\mathcal {C}$ is similar to a multiple elliptic hypergeometric series studied, for example, in [93].

In [61] we have shown that the Abelian blocks ($r=1$) are annihilated by a difference operator which is an elliptic deformation (in the shift operator) of the *q*-hypergeometric operator. We believe that there should exist a similar operator annihilating the more general block given in (3.15), and it would be interesting to determine it.

The 4d holomorphic block (3.15) has the form of an elliptic deformation of a vortex partition function [94], similar to those appearing in [61, 91, 95, 96]. Given the relation between vortex and instanton counting [94, 97–100], it is natural to ask whether (3.15) can be seen as the vortex partition function of a 1/2 BPS codimension 2 theory in $\mathbb {R}^4_{\epsilon _1,\epsilon _2}\times \mathbb {T}^2$. Granted the comment in the previous paragraph, this possibility is also strongly supported by the very well-known fact that partition functions of defect theories obey difference equations [44, 98–102]. Indeed, in the next section we will verify that the elliptic vortex sum in (3.15) equals the $\mathbb {R}^4\times \mathbb {T}^2$ Nekrasov instanton partition function [62–64] of the *U*(*N*) theory with *N* fundamental and anti-fundamental flavors for particular values of the Coulomb branch parameters of the 6d theory.

## 6d Nekrasov partition function

In this section, we compute the instanton partition function on $\mathbb {R}^4_{\epsilon _1,\epsilon _2}\times \mathbb {T}^2$, which can be defined as the generating function of elliptic genera [103, 104] of the instanton moduli space. Our goal is to show that the elliptic Nekrasov instanton partition function reduces to the elliptic vortex partition function (3.15) at specific points in the Coulomb branch. In fact, in analogy with the lower dimensional cases, these should correspond to the points where vortex solutions exist and where the low energy dynamics can be described by a 1/2 BPS codimension 2 theory on $\mathbb {R}^2_\epsilon \times \mathbb {T}^2$.^8^

**Fig. 2:**
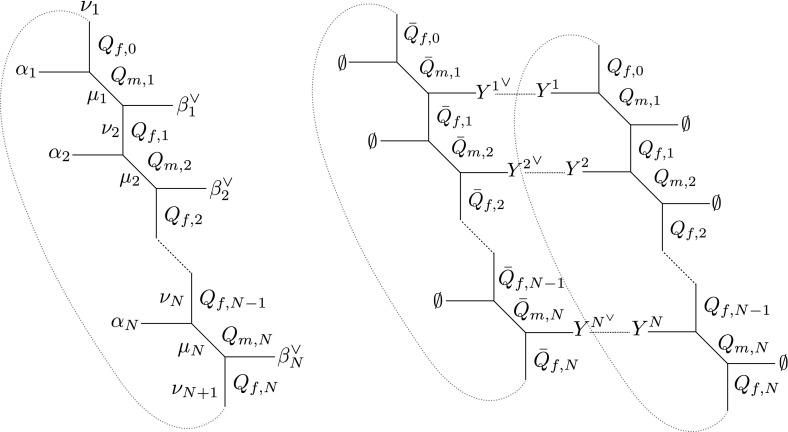
*Left* the (dual) toric diagram of the periodic strip. *Right* gluing two strips

There are diverse methods [62] to compute the supersymmetric partition function we are interested in, such as instanton calculus [4, 5] or topological string methods [106, 107]. We adopt the second perspective. We start by considering an M-theory setup provided by *M* parallel M5-branes wrapped on a torus and probing a transverse $A_{N-1}$ singularity as in [63, 64]4.1Our aim is then to compute the M-theory partition function on such background. The result we are looking for can be found in [63], here we briefly review the points of that construction which are more relevant for our analysis and adapt to our notation. For coincident M5-branes the configuration leads to a 6d (1, 0) superconformal theory. However, one can also consider a deformation away from the superconformal fixed point by separating the M5-branes along a transverse direction, and suspending M2-branes between consecutive M5-branes. The ends of the M2-branes appear as strings from the M5-brane viewpoint, and hence were called $\mathrm{M}_A$-strings in [63] (the $N>1$ generalization of M-strings [108]). The M-theory partition function can be obtained by computing the BPS degeneracies of the states arising from the M2-branes wrapping $\mathbb {T}^2$. Through a chain of dualities, the M-theory background has a dual description in type IIB string theory as the (*p*, *q*)-web [109, 110]4.2where the subindex denotes the (*p*, *q*)-cylinder. The (*p*, *q*)-web is dual to an elliptically fibered toric Calabi–Yau threefold [111, 112]. This geometry can be used to compute IIA topological string amplitudes by using the refined topological vertex [107, 113, 114]. The basic building block of the geometry is given by the periodic strip depicted in Fig. 2 (left), where we have explicitly shown the external Young diagrams and the various Kähler parameters. Because of the periodic identification, all the variables of the strip are subject to the equivalence relation $n\sim n+N$ for any subindex of Kähler parameters or Young diagrams. Notice that there is an additional Kähler parameter ($Q_{f,0}\sim Q_{f,N}$) and internal Young diagram ($\nu _1\sim \nu _{N+1}$) with respect to the uncompactified strip. By choosing the horizontal direction as the preferred one, the sums over the internal diagrams can be performed through the refined version of the method of [115], the main difference being the appearance of an extra infinite product taking into account the multi-covering contributions of the basic holomorphic curves due to the periodic identification. The resulting (normalized) periodic strip amplitude can be written as (11.15)

4.3 $$\begin{aligned}&\frac{\mathcal {K}^{\vec \alpha }_{\vec \beta }( Q_m,Q_f;q,t)}{\mathcal {K}^{\vec \emptyset }_{\vec \emptyset }( Q_m,Q_f;q,t)} =\prod _{a=1}^N\frac{q^{\frac{\Vert \alpha _a\Vert ^2}{2}}t^{\frac{\Vert \beta ^\vee _a\Vert ^2}{2}}{\tilde{Z}}_{\alpha _a}(t,q){\tilde{Z}}_{\beta _a^\vee }(q,t)}{\prod _{k=0}^\infty N_{\beta _a\beta _{a}}(p q'^{k+1};q,t)N_{\alpha _a\alpha _{a}}(q'^{k+1};q,t)}\nonumber \\&\quad \times \frac{\prod _{a,b=1}^N N_{\alpha _{a}\beta _{b}}(p^{\frac{1}{2}}Q_{ab}Q_{m,b};q,t|q')}{\prod _{1\le a\ne b\le N}\prod _{k=0}^\infty N_{\beta _a\beta _{b}}(p q'^{k}Q_{ab}Q_{m,a}^{-1}Q_{m,b};q,t)N_{\alpha _a\alpha _{b}}(q'^{k}Q_{ab};q,t)},\nonumber \\ \end{aligned}$$

with

4.4 $$\begin{aligned} p=qt^{-1},\quad q'=\prod _{k=1}^N Q_{m,k}Q_{f,k},\quad Q_{ab}=\left\{ \begin{array}{ll}\prod _{k=a}^{b-1}Q_{m,k}Q_{f,k},&{} a<b\\ 1,&{}a=b\\ q' Q_{b a}^{-1},&{} a>b\end{array}\right. . \end{aligned}$$

The function $N_{\mu \nu }(Q;q,t|q')$ defined in (11.14) is the elliptic version of the K-theoretic Nekrasov function $N_{\mu \nu }(Q;q,t)$ (11.10). The parameters of the $\Omega$-background are identified as $q=\mathrm{e}^{2\pi \mathrm{i}\epsilon _1}, t=\mathrm{e}^{-2\pi \mathrm{i}\epsilon _2}, q'=\mathrm{e}^{2\pi \mathrm{i}\sigma }$, where $\sigma$ is the elliptic modulus. The 6d theory we are interested in can be engineered by gluing two periodic strips ($M=2$) as in Fig. 2 (right) with the constraint

4.5 $$\begin{aligned} Q_{m,a}Q_{f,a}={\bar{Q}}_{m,a+1}{\bar{Q}}_{f,a}\quad \Rightarrow \quad {\bar{Q}}_{ab}= Q_{ab}{\bar{Q}}_{m,a}{\bar{Q}}_{m,b}^{-1}. \end{aligned}$$

Setting the external legs to empty Young tableaux, we can compute the $\mathbb {R}^4\times \mathbb {T}^2$ Nekrasov instanton partition function for the *U*(*N*) theory with *N* fundamental and anti-fundamental hypers [62]. The details of the gluing are reported in Appendix 6, the final result is^9^

4.6 $$\begin{aligned} \mathcal {Z}^{\mathbb {R}^4\times \mathbb {T}^2}_{\mathrm{inst}}=\sum _{\vec Y}{\tilde{Q}}_B^{| \vec Y| } \prod _{a,b=1}^N\frac{N_{\emptyset Y^b}(A_b^{-1}{\bar{Q}}_a;q,t|q') N_{Y^a\emptyset }(A_a Q_b;q,t|q')}{N_{Y^a Y^b}(A_a A_b^{-1};q,t|q')}, \end{aligned}$$

where we set

4.7 $$\begin{aligned} Q_{ab}=\left\{ \begin{array}{ll}A_a A_b^{-1},&{} a\le b\\ q' A_a A_b^{-1},&{} a>b\end{array}\right. ,\quad Q_{m,a}=A_a Q_a p^{-\frac{1}{2}},\quad {\bar{Q}}_{m,a}=A_a^{-1} {\bar{Q}}_a p^{-\frac{1}{2}}. \end{aligned}$$

Due to $N_{Y^a Y^b}(Q;q,t|0)=N_{Y^a Y^b}(Q;q,t)$, in the decompactification limit $q'\rightarrow 0$ we can recognize in the expression above the $\mathbb {R}^4\times S^1$ Nekrasov instanton partition function upon identifying

4.8 $$\begin{aligned} A_b=\mathrm{e}^{R a_b},\quad Q_b=\mathrm{e}^{R m_b},\quad {\bar{Q}}_b=\mathrm{e}^{R {\bar{m}}_b },\quad {\tilde{Q}}_B= \Lambda ^{\mathrm{5d}}_{\mathrm{inst}}, \end{aligned}$$

where *R* is the scale of the surviving circle, while $a_b, m_b, {\bar{m}}_b, \Lambda ^{\mathrm{5d}}_{\mathrm{inst}}$ are, respectively, the Coulomb branch parameters, the masses of fundamental and anti-fundamental hypers and the 5d instanton parameter.

We now consider a particular specialization of the parameters $A_a$. If we tune

4.9 $$\begin{aligned} A_aQ_a=t^{r_a},\quad r_a\in \mathbb {Z}_{>0},\quad a=1,\ldots ,N, \end{aligned}$$

the numerator of (4.6) yields zero at the box $(r_a+1,1)\in Y^a$, and hence the sum over the Young tableaux is truncated to tableaux with at most $r_a$ rows. When $Y^a$ has at most $r_a$ rows we have

4.10 $$\begin{aligned} N_{Y^a\emptyset }(Q;q,t|q')=\prod _{i=1}^{r_a}\prod _{j=1}^{Y^a_i}\Theta (Q q^{j-1}t^{1-i};q')=\prod _{i=1}^{r_a}\frac{\Gamma (Qq^{Y^a_i}t^{1-i};q,q')}{\Gamma (Qt^{1-i};q,q')}, \end{aligned}$$

where we used $\{\lambda _i-j\}=\{j-1\}$ at fixed *i* and the definition of the $\Theta$-factorial (6.14). Similarly

4.11 $$\begin{aligned} N_{\emptyset Y^a}(Q;q,t|q')=\prod _{i=1}^{r_a}\prod _{j=1}^{Y^a_i}\Theta (Qq^{-j}t^{i};q')=\prod _{i=1}^{r_a}\frac{\Gamma (q q' Q^{-1}q^{Y^a_i}t^{-i};q,q')}{\Gamma (q q' Q^{-1}t^{-i};q,q')}. \end{aligned}$$

When the diagrams are both non-empty, we can use the identity (for $|q|<1$) [117]

4.12 $$\begin{aligned} N_{\mu \nu }(Q;q,t)=\prod _{i,j\ge 1}\frac{(Qq^{\mu _i-\nu _j}t^{j-i+1};q)_\infty }{(Qt^{j-i+1};q)_\infty }\frac{(Qt^{j-i};q)_\infty }{(Qq^{\mu _i-\nu _j}t^{j-i};q)_\infty }, \end{aligned}$$

and the definitions (6.9), (11.14) to write

4.13 $$\begin{aligned} N_{\mu \nu }(Q;q,t|q')=\prod _{i,j\ge 1}\frac{\Gamma (Qt^{j-i+1};q,q')}{\Gamma (Qq^{\mu _i-\nu _j}t^{j-i+1};q,q')}\frac{\Gamma (Qq^{\mu _i-\nu _j}t^{j-i};q,q')}{\Gamma (Qt^{j-i};q,q')}. \end{aligned}$$

Therefore, when $Y^a$ and $Y^b$ have at most $r_a$ and $r_b$ rows, respectively, we have

4.14 $$\begin{aligned} N_{Y^a Y^b}(Q;q,t|q')= & {} \prod _{i=1}^{r_a}\prod _{j=1}^{r_b}\frac{\Gamma (Qt^{j-i+1};q,q')}{\Gamma (Qq^{Y^a_i-Y^b_j}t^{j-i+1};q,q')}\frac{\Gamma (Qq^{Y^a_i-Y^b_j}t^{j-i};q,q')}{\Gamma (Qt^{j-i};q,q')} \nonumber \\&\times N_{Y^a\emptyset }(Qt^{r_b};q,t|q')N_{\emptyset Y^b}(Qt^{-r_a};q,t|q'), \end{aligned}$$

where we have divided the infinite products in four regions, namely $(i,j)\in [1,r_a]\times [1,r_b], (i,j)\in [1,r_a]\times [r_b+1,+\infty ], (i,j)\in [r_a+1,+\infty ]\times [1,r_b], (i,j)\in [r_a+1,+\infty ]\times [r_b+1,+\infty ]$. The first region contributes with the first factor, the second and the third regions yield the elliptic Nekrasov functions with an empty tableaux, while the fourth region does not contribute. Finally, the evaluation of (11.16) at $A_a=Q_a^{-1}t^{r_a}$ yields

4.15 $$\begin{aligned} \mathcal {Z}_{\mathrm{inst}}^{\mathbb {R}^4\times \mathbb {T}^2}=\sum _{\vec Y}{\tilde{Q}}_B^{|\vec Y|}\prod _{a,b=1}^N\frac{\mathcal {Z}^{\mathrm{ad}}_{Y^a Y^b}}{\mathcal {Z}^{\mathrm{ad}}_{\emptyset \emptyset }}\prod _{a=1}^N\frac{\mathcal {Z}^{\mathrm{f}}_{Y^a}\mathcal {Z}^{\bar{\mathrm{f}}}_{Y^a}}{\mathcal {Z}^{\mathrm{f}}_{\emptyset }\mathcal {Z}^{\bar{\mathrm{f}}}_{\emptyset }}, \end{aligned}$$

where

4.16 $$\begin{aligned} \begin{aligned} \mathcal {Z}^{\mathrm{ad}}_{Y^aY^b}&=\prod _{i=1}^{r_a}\prod _{j=1}^{r_b}\frac{\Gamma (t Q_b Q_a^{-1}t^{r_a-r_b+j-i}q^{Y^a_i-Y^b_j};q,q')}{\Gamma (Q_b Q_a^{-1}t^{r_a-r_b+j-i}q^{Y^a_i-Y^b_j};q,q')},\\ \mathcal {Z}^{\mathrm{f}}_{Y^a}\mathcal {Z}^{\bar{\mathrm{f}}}_{Y^a}&=\prod _{b=1}^N\prod _{i=1}^{r_a}\frac{\Gamma (Q_b^{-1}Q_a t^{-r_a+i}q^{-Y^a_i};q,q')}{\Gamma ({\bar{Q}}_b Q_a t^{-r_a+i}q^{-Y^a_i};q,q')}. \end{aligned} \end{aligned}$$

We can now easily identify (4.15) with the elliptic vortex sum in (3.15) provided the following identifications hold^10^

4.17 $$\begin{aligned} \begin{array}{c|c|c|c|c|c|c} \mathbb {R}^2\times \mathbb {T}^2/\mathrm{EVA}~&{}~ q_\tau ~&{}~q_\sigma ~&{}~t~&{}~u_a~&{}~y_a~&{}~q_\tau ^\xi \\ \hline \mathbb {R}^4\times \mathbb {T}^2~&{}~q~&{}~q'~&{}~t~&{}~Q_a{\bar{Q}}_a~&{}~Q_a^{-1}~&{}~{\tilde{Q}}_B~ \end{array}. \end{aligned}$$

Moreover, the specialization of the Coulomb branch parameters/internal momenta encodes the rank *r* of the 4d gauge group/number of screening currents, and it also determines the choice of the 4d block integral/EVA correlator contour through the breaking pattern $r=\sum _{a=1}^{N}r_a$.

## Discussion and outlook

In the special case of the 4-point function ($N=2$) with a single screening current ($r=1$), corresponding to the SQED theory with $N=2$ fundamentals and anti-fundamentals, we have shown in [61] that the 4d holomorphic block (proportional to the elliptic series ${}_2E_1$) satisfies a *q*-difference equation representing an elliptic deformation of the equation satisfied by the ${}_2\phi _1$ *q*-hypergeometric. In *q*-Virasoro theories this corresponds to the fact that the 4-point correlator has a degenerate insertion at level 2, analogously to the very well-known case of (undeformed) Virasoro theories. It is tempting to make an analogous statement for elliptic Virasoro theories, interpreting the elliptic *q*-difference equation as a decoupling equation for the insertion of a degenerate operator. This is certainly true from the gauge theory viewpoint, as we have shown that the Abelian block arises upon the specialization (4.9) $a_1=-m_1-\epsilon _2, a_2=-m_2$ of the elliptic Nekrasov instanton partition function, corresponding in the AGT dictionary to the insertion of a level 2 degenerate external momentum [14]. In order to fully understand this aspect, a study of the representation theory of the EVA is required.

The results of this work summarized in the “triality” (4.17) and the above observations strongly suggest that, in the spirit of the AGT correspondence, generic chiral blocks of the EVA are described by elliptic Nekrasov instanton partition functions. We hope that this 6d AGT relation and the EVA can be a useful tool for studying certain 6d supersymmetric theories and their defects. It would also be interesting to study the 4d/6d/EVA “triality” from the perspective of [119–121].

As we mentioned in the main text, our construction of the EVA can be easily generalized to define an elliptic deformation of the $W_M$ algebra. We expect this extended algebra to be important for studying 4d quiver gauge theories and 6d theories engineered by gluing an arbitrary number of periodic strips. It would be also very interesting to develop a stronger version of the 6d AGT correspondence through the identification of compact space partition functions with non-chiral correlators in QFTs with elliptic Virasoro symmetry, along the lines of [30, 31] for the 5d case.

The EVA may be also interesting from a purely mathematical viewpoint and applications to elliptic integrable systems. The DVA was introduced to understand the symmetry algebra behind Macdonald polynomials, in analogy with the relation between Jack polynomials and singular vectors of the Virasoro algebra. It was eventually understood [122] that the DVA and Macdonald polynomials are naturally related to a more elementary algebra, the trigonometric Ding–Iohara algebra [123]. Elliptic Macdonald functions can be defined as eigenfunctions of the elliptic Macdonald operator. However, their study is much more complicated than in the trigonometric case (see [102, 124] for developments from a gauge theory viewpoint). In [125] an elliptic Ding–Iohara algebra was introduced^11^ and its connection to elliptic Macdonald functions was established. In Appendix 5, we show that the EVA can be realized on a tensor product of two Fock representations of the elliptic Ding–Iohara algebra. It is then natural to ask whether elliptic Macdonald functions can be studied by means of the EVA, perhaps through their correspondence with some kind of singular vectors. This perspective may eventually lead to a neat integral representation of elliptic Macdonald functions, as in [22, 127] for the trigonometric case.

Finally, in [128] the important role of the trigonometric Ding–Iohara algebra for the 5d AGT relation was extensively discussed. It was conjectured (and proved in the Abelian case) that topological string amplitudes on the strip, the basic building block for the 5d Nekrasov instanton partition function, can be computed as matrix elements of a vertex operator intertwining representations of the trigonometric Ding–Iohara algebra. Given the relation among the 6d Nekrasov instanton partition function, the EVA and the elliptic Ding–Iohara algebra found in this work, it would be interesting to understand whether periodic strip amplitudes have a similar interpretation.^12^

*Comment added* Clavelli–Shapiro trace technique [130] allows torus correlators in *q*-$W_M$ algebras to be interpreted as sphere correlators in elliptic $W_M$ algebras. The advantage of this perspective is that the latter are usually easier to handle. This relation between trigonometric and elliptic algebras is related to the fiber/base duality in the context of 5d or 6d theories arising from toric Calabi–Yau threefolds with a periodic direction discussed in this paper. However, this duality does not imply that all the elliptic $W_M$ algebra observables can be recast in terms of *q*-$W_M$ algebra ones. This is, for instance, the case of elliptic torus correlators, which should be interesting for doubly compactified toric geometries. Moreover, it seems to be a non-trivial fact that the elliptic deformation leads to a well-defined associative algebra.

## A Elliptic functions

In this appendix, we collect useful definitions and properties of some elliptic functions used in the main text. We refer to [135] for further details. We start by defining the (infinite) *q*-factorial

6.1 $$\begin{aligned} (x;q)_\infty =\mathrm{e}^{-\mathrm{Li}_2(x;q)},\quad \mathrm{Li}_2(x;q)=\sum _{k\ge 1}\frac{x^k}{k(1-q^k)}. \end{aligned}$$

In the region $|q|<1$, it has the compact product representation

6.2 $$\begin{aligned} (x;q)_\infty =\prod _{k\ge 0}(1-q^k x), \end{aligned}$$

which can be extended to the domain $|q|>1$ through

6.3 $$\begin{aligned} (x;q)_\infty = \frac{1}{\prod _{k\ge 1}(1-q^{-k} x)}. \end{aligned}$$

The Jacobi Theta function that we use is defined by

6.4 $$\begin{aligned} \Theta (x;q)=(x;q)_\infty (q x^{-1};q)_\infty =\mathrm{e}^{-\sum _{n\ne 0}\frac{x^n}{n(1-q^n)}}. \end{aligned}$$

From this simple expression we can realize that throughout this paper we will consider exponentials of infinite sums running in both directions. These series can be organized by replacing the expansion parameter $x^{n}$ with $\ell ^{|n|} x^{n}$, expanding in $\ell$ and then letting $\ell \rightarrow 1$. This is, for instance, how one can verify on a computer Jacobi’s triple product identity

6.5 $$\begin{aligned} (x;q)_\infty (qx^{-1};q)_\infty =\frac{1}{(q;q)_\infty }\sum _{n\in \mathbb {Z}}(-1)^n q^{n(n-1)/2}x^n. \end{aligned}$$

Useful properties of Theta functions are ($m\in \mathbb {Z}_{\ge 0}$)

6.6 $$\begin{aligned} \frac{\Theta (q^m x;q)}{\Theta (x;q)}=(-x q^{(m-1)/2})^{-m},\quad \frac{\Theta (q^{-m}x;q)}{\Theta (x;q)}=(-x^{-1}q^{(m+1)/2})^{-m}. \end{aligned}$$

The double (infinite) *q*-factorial is defined by

6.7 $$\begin{aligned} (x;p,q)_\infty =\prod _{j,k\ge 0}(1-p^j q^k x),\quad |p|,|q|<1, \end{aligned}$$

and it can be extended to other regions by using the representation

6.8 $$\begin{aligned} (x;p,q)_\infty =\mathrm{e}^{-\mathrm{Li}_3(x;p,q)},\quad \mathrm{Li}_3(x;p,q)=\sum _{k\ge 1}\frac{x^k}{k(1-p^k)(1-q^k)}. \end{aligned}$$

The elliptic Gamma function is defined by

6.9 $$\begin{aligned} \Gamma (x;p,q)=\frac{(p q x^{-1};p,q)_\infty }{(x;p,q)_\infty }=\mathrm{e}^{\sum _{k\ne 0}\frac{x^k}{k(1-p^k)(1-q^k)}}=\mathrm{e}^{\sum _{k>0}\frac{(q^{-1/2}p^{-1/2}x)^k}{k(q^{k/2}-q^{-k/2})(p^{k/2}-p^{-k/2})}}. \end{aligned}$$

Assuming $|p|, |q|<1$, it has zeros and poles at

6.10 $$\begin{aligned} \text {zeros}:~ x=p^{m+1}q^{n+1},\quad \text {poles}:~ x=p^{-m}q^{-n},\quad m,n\in \mathbb {Z}_{\ge 0}. \end{aligned}$$

Useful properties of the elliptic Gamma function are ($m,n\in \mathbb {Z}_{\ge 0}$)

6.11 $$\begin{aligned}&\mathrm{Reflection:}~~\Gamma (x;p,q)\Gamma (p q x^{-1};p,q)=1, \end{aligned}$$

6.12 $$\begin{aligned}&\mathrm{Shift:}~~\begin{aligned}&\frac{\Gamma (p^m q^n x;p,q)}{\Gamma (x;p,q)}=(-x p^{(m-1)/2}q^{(n-1)/2})^{-m n}\Theta (x;p,q)_n\Theta (x;q,p)_m,\\&\frac{\Gamma (p^m q^{-n} x;p,q)}{\Gamma (x;p,q)}=(-x p^{(m-1)/2}q^{-(n+1)/2})^{m n}\frac{\Theta (x;q,p)_m}{\Theta (p q x^{-1};p,q)_n}, \end{aligned} \end{aligned}$$

6.13 $$\begin{aligned}&\mathrm{Residues:}~~\text {Res}_{x=y p^m q^n}\frac{\Gamma (y x^{-1};p,q)}{x}\nonumber \\&\quad =\text {Res}_{x=1}\Gamma (x;p,q)~\frac{(-p q~q^{(n-1)/2}p^{(m-1)/2})^{m n}}{\Theta (p q;p,q)_n\Theta (p q;q,p)_m}. \end{aligned}$$

Here we introduced the $\Theta$-factorial

6.14 $$\begin{aligned} \Theta (x;p,q)_n&=\frac{\Gamma (q^n x;p,q)}{\Gamma (x;p,q)}=\prod _{k=0}^{n-1}\Theta (x q^k;p), \end{aligned}$$

6.15 $$\begin{aligned} \Theta (x;p,q)_{-n}&=\Theta (q^{-n}x;p,q)_{n}^{-1}. \end{aligned}$$

Notice that in the limit $p\rightarrow 0$ the $\Theta$-factorial reduces to the *q*-factorial

6.16 $$\begin{aligned} \Theta (x;0,q)_n=(x;q)_n=\prod _{k=0}^{n-1}(1-q^k x), \end{aligned}$$

because

6.17 $$\begin{aligned} \Theta (x;0)=1-x. \end{aligned}$$

## B Free boson tools

The master formula when manipulating with bosonic oscillators is the BCH formula. Given two operators $\mathsf{A}, \mathsf{B}$ such that  for some constant $c\in \mathbb {C}$, the BCH formula reduces to

7.1 $$\begin{aligned} \mathrm{e}^\mathsf{A}\mathrm{e}^\mathsf{B}=\mathrm{e}^\mathsf{B}\mathrm{e}^\mathsf{A}\mathrm{e}^{c},\quad \mathrm{e}^{-\mathsf{A}}\mathsf{B}\mathrm{e}^\mathsf{A}=\mathsf{B}-[\mathsf{A},\mathsf{B}]. \end{aligned}$$

Let us now consider an algebra generated by the operators $\{\mathsf{a}_n, n\in \mathbb {Z}\backslash \{0\} \}$ with the defining relations

7.2

We can construct the following vertex operators

7.3 $$\begin{aligned} \mathsf{V}_i=\; :\mathrm{e}^{\mathsf{v}_i}:,\quad \mathsf{v}_i=\sum _{n\ne 0}v_{i,n}\mathsf{a}_n, \end{aligned}$$

where $v_{i,n}$ are generic complex numbers and the normal ordering symbol  :   :  means all the positive modes are moved to the right of all the negative modes. If we denote by $[~]_\pm$ the positive and negative mode parts, we can then write

7.4 $$\begin{aligned} \mathsf{V}_i=[\mathsf{V}_i]_-[\mathsf{V}_i]_+, \quad [\mathsf{V}_i]_\pm =\mathrm{e}^{[\mathsf{v}_i]_\pm }. \end{aligned}$$

In the main text, we have to compute correlators containing expressions such as

7.5 $$\begin{aligned} \prod _{i=1}^M \mathsf{V}_{i}=[\mathsf{V}_1]_-[\mathsf{V}_1]_+[\mathsf{V}_2]_-[\mathsf{V}_2]_+~\cdots ~ [\mathsf{V}_M]_-[\mathsf{V}_M]_+. \end{aligned}$$

To this end, we bring all the $[\mathsf{V}_i]_+$s to the right of all the $[\mathsf{V}_j]_-$s. The first, $[\mathsf{V}_1]_+$, has to cross all the $[\mathsf{V}_i]_-$ with $i=2,\ldots ,M$, while it commutes with all the $[\mathsf{V}_i]_+$s. In the process it produces $\mathrm{e}^{[[\mathsf{v}_1]_+,[\mathsf{v}_2]_-]}\cdots \mathrm{e}^{[[\mathsf{v}_1]_+,[\mathsf{v}_M]_-]}$ due to (7.1). The second, $[V_2]_+$, has to cross all the $[\mathsf{V}_i]_-$ with $i=3,\ldots ,M$, and so on. Eventually, we get

7.6 $$\begin{aligned} \prod _{i=1}^M \mathsf{V}_{i}=\;:\prod _{i=1}^M \mathsf{V}_i:\times \prod _{1\le i<j\le M}\mathrm{e}^{[[\mathsf{v}_i]_+,[\mathsf{v}_j]_-]}. \end{aligned}$$

## C Free boson representation of the EVA

In this section we give the proof that the current (2.9) satisfies the defining relation (2.1) of the EVA.^13^ Let us start by computing the “OPE”

8.1 $$\begin{aligned} \mathsf{T}(z)\mathsf{T}(w)&=\Lambda _+(z)\Lambda _+(w)+\Lambda _+(z)\Lambda _-(w)+\Lambda _-(z)\Lambda _+(w)+\Lambda _-(z)\Lambda _-(w)\nonumber \\&=\;:\Lambda _+(z)\Lambda _+(w):f_{+,+}(w/z)^{-1}+:\Lambda _+(z)\Lambda _-(w):f_{+,-}(w/z)^{-1}\nonumber \\&~~~+:\Lambda _-(z)\Lambda _+(w):f_{-,+}(w/z)^{-1}+:\Lambda _-(z)\Lambda _-(w):f_{-,-}(w/z)^{-1}. \end{aligned}$$

Using (2.13) we have

8.2 $$\begin{aligned} f(w/z)\mathsf{T}(z)\mathsf{T}(w)&=\;:\Lambda _+(z)\Lambda _+(w):+:\Lambda _+(z)\Lambda _-(w):\gamma (p^{1/2}w/z)\nonumber \\&~~~+:\Lambda _-(z)\Lambda _+(w):\gamma (p^{-1/2}w/z)+:\Lambda _-(z)\Lambda _-(w):, \end{aligned}$$

and similarly for $\mathsf{T}(w)\mathsf{T}(z)f(z/w)$ which is obtained by exchanging $z\leftrightarrow w$. Subtracting the two equalities we get

8.3 $$\begin{aligned}&f(w/z)\mathsf{T}(z)\mathsf{T}(w)-\mathsf{T}(w)\mathsf{T}(z)f(z/w)=\; :\Lambda _+(z)\Lambda _-(w):(\gamma (p^{1/2}w/z)\nonumber \\&\quad -\gamma (p^{-1/2}z/w))+:\Lambda _-(z)\Lambda _+(w):(\gamma (p^{-1/2}w/z)-\gamma (p^{-1/2}z/w)). \end{aligned}$$

Now using (2.15) we have ($\kappa =\frac{\Theta (q;q')\Theta (t^{-1};q')}{(q';q')^2_\infty \Theta (p,;q')}$)

8.4 $$\begin{aligned}&f(w/z)\mathsf{T}(z)\mathsf{T}(w)-\mathsf{T}(w)\mathsf{T}(z)f(z/w)\nonumber \\&\quad =\; -\kappa :\Lambda _+(z)\Lambda _-(w):(\delta (p w/z)-\delta (w/z))\nonumber \\&\qquad -\kappa :\Lambda _-(z)\Lambda _+(w):(\delta (w/z)-\delta (p^{-1}w/z))\nonumber \\&\quad =-\kappa :\Lambda _+(z)\Lambda _-(p^{-1}z):\delta (p w/z)\nonumber \\&\qquad +\kappa :\Lambda _-(z)\Lambda _+(p z):\delta (p^{-1}w/z), \end{aligned}$$

and we can use (2.12) to conclude the proof. This is the final result, but the relation (2.15) needs to be considered more carefully. First of all, one may naively conclude that

8.5 $$\begin{aligned} \gamma (x)-\gamma (x^{-1})=0, \end{aligned}$$

because

8.6 $$\begin{aligned} \gamma (x)=\frac{\Theta (p^{1/2}q^{-1}x;q')\Theta (p^{-1/2}q x;q')}{\Theta (p^{1/2}x;q')\Theta (p^{-1/2}x;q')}=\gamma (x^{-1}), \end{aligned}$$

where in the last equality we used the property

8.7 $$\begin{aligned} \Theta (x^{-1};q')=\Theta (q' x;q' )=-x^{-1} \Theta (x;q'). \end{aligned}$$

This is the elliptic analogue of the identity

8.8 $$\begin{aligned} \frac{1}{1-x}=-\frac{x^{-1}}{1-x^{-1}}, \end{aligned}$$

to which it reduces in the limit $q'\rightarrow 0$. However, we have to remember that the difference $\gamma (x)-\gamma (x^{-1})$ is coming from subtracting the operator $\mathsf{T}(w)\mathsf{T}(z)f(z/w)$ from the operator $\mathsf{T}(z)\mathsf{T}(w)f(w/z)$, and hence non-trivial contact terms, signaled by $\delta$ functions, may arise in the process because a radial ordering prescription is needed. In other words, the difference $\gamma (x)-\gamma (x^{-1})$ must be treated in the sense of hyperfunctions (see, for example, [131]). All in all, the problem is to find a concrete formula for the hyperfunction $\gamma (x)-\gamma (x^{-1})$. Before considering the elliptic case, it might be useful to recall how $\delta$ function terms arise in the *q*-Virasoro limit (see, for example, appendix of [83]), in which case the $\gamma (x)$ function is substituted by the $q'\rightarrow 0$ limit of the one appearing here

8.9 $$\begin{aligned} \gamma (x)\mathop {\rightarrow }\limits ^{q'\rightarrow 0}{\tilde{\gamma }}(x)=\frac{(1-p^{1/2}q^{-1}x)(1-p^{-1/2}q x)}{(1-p^{1/2}x)(1-p^{-1/2}x)}. \end{aligned}$$

First of all, the identity (8.8) must now be replaced/modified by

8.10 $$\begin{aligned} \delta (x)=\frac{1}{1-x}+\frac{x^{-1}}{1-x^{-1}}, \end{aligned}$$

which can be easily proved by series expanding the two terms in the r.h.s. separately. Now we focus on the factor $(1-p^{1/2}x)$ in the denominator of ${\tilde{\gamma }}(x)$, which will be replaced by

8.11 $$\begin{aligned} \frac{1}{1-p^{1/2}x}=\delta (p^{1/2}x)-\frac{p^{-1/2}x^{-1}}{1-p^{-1/2}x^{-1}}, \end{aligned}$$

and similarly for the factor $(1-p^{1/2}x^{-1})$ in the denominator of ${\tilde{\gamma }}(x^{-1})$

8.12 $$\begin{aligned} \frac{p^{1/2}x^{-1}}{1-p^{1/2}x^{-1}}=\delta (p^{-1/2}x)-\frac{1}{1-p^{-1/2}x}. \end{aligned}$$

Now we can take the difference ${\tilde{\gamma }}(x)-{\tilde{\gamma }}(x^{-1})$ naively, and only the $\delta$ function terms will survive

8.13 $$\begin{aligned} {\tilde{\gamma }}(x)-{\tilde{\gamma }}(x^{-1})=-\frac{(1-q)(1-t^{-1})}{(1-p)}(\delta (p^{1/2}x)-\delta (p^{-1/2}x)). \end{aligned}$$

In the elliptic case one can repeat exactly the same steps, the crucial point being to find a $\delta$ function representation involving Theta functions which can be used to replace/modify the identity (8.7). This representation is

8.14 $$\begin{aligned} \delta (x)=\frac{(q';q')^2_\infty }{\Theta (x;q')}+\frac{(q';q')^2_\infty x^{-1}}{\Theta (x^{-1};q')}, \end{aligned}$$

which is given in [125] (*Lemma* 3.5). Now we can use this representation to replace the factor $\Theta (p^{1/2}x;q')$ in the denominator of $\gamma (x)$ with

8.15 $$\begin{aligned} \frac{1}{\Theta (p^{1/2}x;q')}=\frac{\delta (p^{1/2}x)}{(q';q')^2_\infty }-\frac{p^{-1/2}x^{-1}}{\Theta (p^{-1/2}x^{-1};q')}, \end{aligned}$$

and similarly for the factor $\Theta (p^{1/2}x^{-1};q')$ in the denominator of $\gamma (x^{-1})$

8.16 $$\begin{aligned} \frac{p^{1/2}x^{-1}}{\Theta (p^{1/2}x^{-1};q')}=\frac{\delta (p^{-1/2}x)}{(q';q')^2_\infty }-\frac{1}{\Theta (p^{-1/2}x;q')}. \end{aligned}$$

This prescription defines the hyperfunction $\gamma (x)-\gamma (x^{-1})$ in (2.15), which is not identically zero due to contact terms.

## D Screening current of the EVA

In this appendix, we provide the explicit verification of (2.16) using (2.9), (2.17). We set

9.1 $$\begin{aligned} \Lambda _\sigma (z)=\; :\mathrm{e}^{\lambda _\sigma (z)}: [\Lambda _\sigma (z)]_0,\quad \mathsf{S}(w)=\; :\mathrm{e}^{\mathsf{s}(w)}: [\mathsf{S}(w)]_0, \end{aligned}$$

where $[~]_{+,-,0}$ denotes the positive, negative or zero mode part. Then, we have

9.2 $$\begin{aligned}{}[\Lambda _\sigma (z),\mathsf{S}(w)]=\; :\Lambda _\sigma (z)\mathsf{S}(w):\left( t^\sigma \mathrm{e}^{[[\lambda _\sigma (z)]_+,[\mathsf{s}(w)]_-]}-\mathrm{e}^{[[\mathsf{s}(w)]_+,[\lambda _\sigma (z)]_-]}\right) . \end{aligned}$$

Since

9.3 $$\begin{aligned}{}[[\lambda _\sigma (z)]_+,[\mathsf{s}(w)]_-]&=\sigma \sum _{n>0}\frac{(p^{\frac{\sigma }{2}}w z^{-1})^n(t^{\frac{n}{2}}-t^{-\frac{n}{2}})}{n(1-q'^n)}\nonumber \\&+\sigma \sum _{n>0}\frac{(q' p^{-\frac{\sigma }{2}}z w^{-1})^n(t^{-\frac{n}{2}}-t^{\frac{n}{2}})}{n(1-q'^n)},\nonumber \\ [[\mathsf{s}(w)]_+,[\lambda _\sigma (z)]_-]&=-\sigma \sum _{n>0}\frac{(p^{-\frac{\sigma }{2}}z w^{-1})^n(t^{\frac{n}{2}}-t^{-\frac{n}{2}})}{n(1-q'^n)}\nonumber \\&-\sigma \sum _{n>0}\frac{(q' p^{\frac{\sigma }{2}}w z^{-1})^n(t^{-\frac{n}{2}}-t^{\frac{n}{2}})}{n(1-q'^n)}, \end{aligned}$$

we get

9.4 $$\begin{aligned} \displaystyle&[\Lambda _\sigma (z),\mathsf{S}(w)]=\;:\Lambda _\sigma (z)\mathsf{S}(w):F_\sigma (p^{\frac{\sigma }{2}} t^{\frac{1}{2}}w z^{-1}), \end{aligned}$$

where

9.5 $$\begin{aligned} F_\sigma (x)=t^\sigma \frac{\Theta (t^{-1} x;q')^\sigma }{\Theta (x;q')^\sigma }-\frac{\Theta (t x^{-1};q')^\sigma }{\Theta (x^{-1};q')^\sigma }. \end{aligned}$$

This function should be interpreted again as a hyperfunction (otherwise it is identically zero). Then,

9.6 $$\begin{aligned} F_+(x)=t\frac{\Theta (t^{-1}x;q')}{(q';q')^2_\infty }\delta (x),\quad F_-(x)=t^{-1}\frac{\Theta (x;q')}{(q';q')^2_\infty }\delta (x t^{-1}), \end{aligned}$$

where we used the representation (8.14). We can therefore write

9.7 $$\begin{aligned}{}[\mathsf{T}(z),\mathsf{S}(w)]=t^\frac{1}{2}\frac{\Theta (t^{-1};q')}{(q';q')^2_\infty }\sum _{\sigma }~\sigma :\Lambda _\sigma (q^{\frac{\sigma }{2}}w)\mathsf{S}(w):t^{\frac{\sigma }{2}}\delta (q^{\frac{\sigma }{2}}w z^{-1}). \end{aligned}$$

It is now easy to verify that

9.8 $$\begin{aligned} :\Lambda _{+}(q^{\frac{1}{2}}w)\mathsf{S}(w):t^{\frac{1}{2}}=\frac{\mathsf{X}(q^{\frac{1}{2}}w)}{w},\quad :\Lambda _{-}\left( q^{-\frac{1}{2}}w\right) \mathsf{S}(w):t^{-\frac{1}{2}}=\frac{\mathsf{X}\left( q^{-\frac{1}{2}}w\right) }{w}, \end{aligned}$$

where

9.9 $$\begin{aligned} \frac{\mathsf{X}(w)}{w}=\; :\mathrm{e}^{-\sum _{n\ne 0}\frac{\left( q^{n/2}p^{-n}+q^{-n/2}\right) }{(1+p^{-n})\left( q^{n/2}-q^{-n/2}\right) (1-q'^{|n|})}(w^{-n}\alpha _n-w^n\beta _n)}:\mathrm{e}^{\sqrt{\beta }\mathsf{Q}}w^{\sqrt{\beta }\mathsf{P}}, \end{aligned}$$

so that

9.10 $$\begin{aligned}{}[\mathsf{T}(z),\mathsf{S}(w)]=\left( q^{\frac{1}{2}}-q^{-\frac{1}{2}}\right) t^{\frac{1}{2}}\frac{\Theta (t^{-1};q')}{(q';q')^2_\infty }\frac{\mathrm{d}}{\mathrm{d}_q w}\delta (w z^{-1})\mathsf{X}(w). \end{aligned}$$

## E Relation with the elliptic Ding–Iohara algebra

The Ding–Iohara algebra [132] is an associative unital (functional) $\mathbb {C}$-algebra endowed with a coproduct $\Delta$ (in fact, a Hopf algebra) and defined by a matrix of analytic structure functions $g_{i j}(z)$ satisfying the property $g_{i j}(z)=g_{j i}(z^{-1})^{-1}$. It provides a generalization of the Drinfeld realization of quantum affine algebras, giving rise to standard examples for particular choices of the structure matrix.

By considering a single structure function of elliptic type

10.1 $$\begin{aligned} g(z)=\frac{\Theta (q z;q')\Theta (t^{-1}z;q')\Theta (p^{-1}z;q')}{\Theta (q^{-1}z;q')\Theta (t z;q')\Theta (p z;q')}, \end{aligned}$$

the author of [125]^14^ introduced the elliptic Ding–Iohara algebra $\mathcal {U}(q,t,q')$ generated by the coefficients of the currents

10.2 $$\begin{aligned} x^\pm (z)=\sum _{n}x^\pm _n z^{-n},\quad \psi ^\pm (z)=\sum _{n}\psi ^\pm _n z^{-n}, \quad n\in \mathbb {Z}, \end{aligned}$$

and the invertible central element $\gamma ^{1/2}$, subject to the following defining relations

10.3 $$\begin{aligned} \begin{aligned} {[}\psi ^\pm (z),\psi ^\pm (w)]&=0,\\ \psi ^+(z)\psi ^-(w)&=\psi ^-(w)\psi ^+(z)\frac{g(\gamma z w^{-1})}{g(\gamma ^{-1}z w^{-1})},\\ \psi ^\rho (z)x^\sigma (w)&=x^\sigma (w)\psi ^\rho (z)g(\gamma ^{\rho \sigma \frac{1}{2}}z w^{-1})^\sigma ,\quad (\sigma ,\rho )\in \{\pm ,\pm \},\\ x^\pm (z)x^\pm (w)&=x^\pm (w)x^\pm (z)g(z w^{-1})^{\pm 1},\\ [x^+(z),x^-(w)]&=\frac{\Theta (q;q')\Theta (t^{-1};q')}{(q';q')_\infty ^2\Theta (p;q')}\left( \delta \left( \gamma \frac{w}{z}\right) \psi ^+(\gamma ^{\frac{1}{2}}w)\right. \\&\left. \quad -\delta \left( \gamma ^{-1}\frac{w}{z}\right) \psi ^-(\gamma ^{-\frac{1}{2}}w)\right) . \end{aligned} \end{aligned}$$

The coproduct reads

10.4

where .

We now show that, using the following level 1^15^ Fock representation $\rho _y$ ($y\in \mathbb {C^\times }$) of $\mathcal {U}(q,t,q')$ ([125], *Theorem* 1.2)

10.5 $$\begin{aligned} \rho _y(\gamma ^{\pm \frac{1}{2}})= & {} p^{\mp \frac{1}{4}},\quad \rho _y(\psi ^\pm (z))=\varphi ^\pm (z),\quad \rho _y(x^+(z))=y\eta (z),\nonumber \\ \rho _y(x^-(z))= & {} y^{-1}\xi (z), \end{aligned}$$

where^16^

10.6 $$\begin{aligned} \begin{aligned} \varphi ^\pm (z)&=[\varphi (z)]_\pm ,\\ \varphi (z)&\!=\!\;:\mathrm{e}^{\sum _{n\ne 0}\frac{(1-t^n)(p^{-|n|/2}-p^{|n|/2})p^{-|n|/4}z^{-n}}{n(1-q'^{|n|})}\mathsf{a}_n}\\&\quad \qquad \mathrm{e}^{-\sum _{n\!\ne \! 0}\frac{(1-t^{-n})(p^{-|n|/2}-p^{|n|/2})p^{|n|/4}q'^{|n|}z^{n}}{n(1-q'^{|n|})}\mathsf{b}_n}:,\\ \eta (z)&=\; :\mathrm{e}^{-\sum _{n\ne 0}\frac{(1-t^n)z^{-n}}{n(1-q'^{|n|})}\mathsf{a}_n}\mathrm{e}^{-\sum _{n\ne 0}\frac{(1-t^{-n})q'^{|n|}z^{n}}{n(1-q'^{|n|})}\mathsf{b}_n}:,\\ \xi (z)&=\; :\mathrm{e}^{\sum _{n\ne 0}\frac{(1-t^n)p^{-|n|/2}z^{-n}}{n(1-q'^{|n|})}\mathsf{a}_n}\mathrm{e}^{\sum _{n\ne 0}\frac{(1-t^{-n})p^{|n|/2}q'^{|n|}z^{n}}{n(1-q'^{|n|})}\mathsf{b}_n}:,\\ [\mathsf{a}_m,\mathsf{a}_n]&=m\frac{(1-q'^{|m|})(1-q^{|m|})}{1-t^{|m|}}\delta _{m+n,0},\quad m,n\in \mathbb {Z}\backslash \{0\},\\ [\mathsf{b}_m,\mathsf{b}_n]&=m\frac{(1-q'^{|m|})(1-q^{|m|})}{(p q')^{|m|}(1-t^{|m|})}\delta _{m+n,0},\quad [\mathsf{a}_m,\mathsf{b}_n]=0,\quad m,n\in \mathbb {Z}\backslash \{0\}, \end{aligned} \end{aligned}$$

we can give a representation of the EVA algebra defined in (2.1) through the tensor product representation $\rho _{y_1}\otimes \rho _{y_2}$. Our derivation follows the analogous construction of [122], where it is shown how to realize the *q*-$W_M$ algebra of [22, 23] starting from the trigonometric Ding–Iohara algebra, the $q'\rightarrow 0$ limit of $\mathcal {U}(q,t,q')$. To begin with, we define the dressed current

10.7 $$\begin{aligned} {t}(z)=\alpha ^-(z)x^+(z)\alpha ^+(z), \end{aligned}$$

where the currents $\alpha ^\pm (z)$ are defined by means of the modes of $\psi ^\pm (z)$ as follows. In analogy with [122] and having in mind the Fock representation (10.5), we set

10.8 $$\begin{aligned} \begin{aligned} \psi ^\pm (z)&=\psi _0^\pm \mathrm{e}^{\pm \sum _{n>0}\Psi _{\pm n}\gamma ^{n/2}z^{\mp n}}\mathrm{e}^{\mp \sum _{n>0}\Psi '_{\pm n}\gamma ^{-n/2}z^{\pm n}},\quad [\psi ^+_0,\psi _0^-]=0,\\ [\Psi _m,\Psi _n]&=\frac{(1-q^{-m})(1-t^m)(1-p^m)}{m(1-q'^{|m|})}(\gamma ^m-\gamma ^{-m})\gamma ^{-|m|}\delta _{m+n,0},\\ [\Psi '_m,\Psi '_n]&=\frac{q'^{|m|}(1-q^{-m})(1-t^m)(1-p^m)}{m(1-q'^{|m|})} (\gamma ^m-\gamma ^{-m})\gamma ^{|m|}\delta _{m+n,0}, \end{aligned} \end{aligned}$$

and take

10.9 $$\begin{aligned} \alpha ^\pm (z)=\mathrm{e}^{\pm \sum _{n>0}\frac{z^{\mp n}}{\gamma ^{n}-\gamma ^{-n}}\Psi _{\pm n}}\mathrm{e}^{\pm \sum _{n>0}\frac{z^{\pm n}}{\gamma ^{n}-\gamma ^{-n}}\Psi '_{\pm n}}. \end{aligned}$$

Notice that the action of the coproduct on $\Psi _n, \Psi '_n$ reads

10.10

while the $\rho _y$ representation is given by $\rho _y(\psi _0^\pm )=1$ and

10.11 $$\begin{aligned} \rho _y(\Psi _n)= & {} \frac{(1-t^n)(p^{-|n|/2}-p^{|n|/2})}{|n|(1-q'^{|n|})}\mathsf{a}_n,\nonumber \\ \rho _y(\Psi '_n)= & {} \frac{q'^{|n|}(1-t^{-n})(p^{-|n|/2}-p^{|n|/2})}{|n|(1-q'^{|n|})}\mathsf{b}_n. \end{aligned}$$

We now consider the twofold tensor product representation

10.12 $$\begin{aligned} \rho _{y_1,y_2}^{(2)}=\rho _{y_1}\otimes \rho _{y_2}\circ \Delta , \end{aligned}$$

and we want to compute

10.13 $$\begin{aligned} \rho _{y_1,y_2}^{(2)}({ t}(z))=\sum _{i=1,2}y_i\Lambda _i(z). \end{aligned}$$

Notice that we are constructing a level 2 representation since $\rho ^{(2)}_{y_1,y_2}(\gamma ^{\pm \frac{1}{2}})=p^{\mp \frac{1}{2}}$. In particular, we have

10.14 $$\begin{aligned} \begin{aligned} \rho ^{(2)}_{y_1,y_2}(\Psi _n)&=\sum _{i=1,2}\rho ^{(2)}_{y_1,y_2,i}(\Psi _n),\\ \rho ^{(2)}_{y_1,y_2,i}(\Psi _n)&=-\frac{(1-t^n)(1-p^{-|n|})p^{\frac{|n|}{2}(2-i+1)}}{|n|(1-q'^{|n|})}\mathsf{a}_{n,i},\\ \rho ^{(2)}_{y_1,y_2}(\Psi '_n)&=\sum _{i=1,2}\rho ^{(2)}_{y_1,y_2,i}(\Psi '_n),\\ \rho ^{(2)}_{y_1,y_2,i}(\Psi '_n)&=\frac{q'^{|n|}(1-t^{-n})(1-p^{|n|})p^{-\frac{|n|}{2}(2-i+1)}}{|n|(1-q'^{|n|})}\mathsf{b}_{n,i}, \end{aligned} \end{aligned}$$

where the subindex *i* denotes that the operators are in the *i* ^th^ tensor component. Therefore,

10.15 $$\begin{aligned} \begin{aligned} \rho _{y_1,y_2}^{(2)}(\alpha ^+(z))&=\prod _{i=1,2}\lambda ^+_{i}(z),\\ \lambda ^+_i(z)&=\mathrm{e}^{\sum _{n>0}\frac{z^{-n}}{p^{-n}-p^{n}}\rho _{y_1,y_2,i}^{(2)}(\Psi _n)}\mathrm{e}^{\sum _{n>0}\frac{z^{n}}{p^{-n}-p^{n}}\rho _{y_1,y_2,i}^{(2)}(\Psi '_{n})},\\ \rho _{y_1,y_2}^{(2)}(\alpha ^-(z))&=\prod _{i=1,2}\lambda ^-_{i}(z),\\ \lambda ^-_i(z)&=\mathrm{e}^{-\sum _{n>0}\frac{z^n}{p^{-n}-p^{n}}\rho ^{(2)}_{y_1,y_2,i}(\Psi _{-n})}\mathrm{e}^{-\sum _{n>0}\frac{z^{-n}}{p^{-n}-p^{n}}\rho ^{(2)}_{y_1,y_2,i}(\Psi '_{-n})}. \end{aligned} \end{aligned}$$

We also have

10.16

and hence

10.17 $$\begin{aligned} \Lambda _i(z)=\prod _{j=1,2}\lambda ^-_j(z){\tilde{\Lambda }}_i(z)\prod _{k=1,2}\lambda ^+_k(z). \end{aligned}$$

Notice that . Finally, we can verify that (2.10) is satisfied with $\Lambda _{+}(z)\rightarrow \Lambda _{1}(z), \Lambda _{-}(z)\rightarrow \Lambda _{2}(z)$, namely

10.18 $$\begin{aligned} \Lambda _{1,2}(z)\Lambda _{1,2}(w)=\; :\Lambda _{1,2}(z)\Lambda _{1,2}(w):f_{\pm ,\pm }(w z^{-1})^{-1}, \end{aligned}$$

which proves that the current $\rho _{1,1}^{(2)}({ t}(z))$ gives a representation of the EVA algebra. For completeness, let us work out explicitly the case of $\Lambda _{1}(z)\Lambda _{1}(w)$, the others can be treated similarly. The first tensor component in the above representation of $\Lambda _{1}(z)\Lambda _{1}(w)$ arises from

10.19 $$\begin{aligned}&\lambda ^-_1(z)\eta (z)\lambda _1^+(z)\lambda _1^-(w)\eta (w)\lambda ^+_1(w)\nonumber \\&\quad =\lambda ^-_1(z)[\eta (z)]_-\Big ([\eta (z)]_+\lambda _1^+(z)\lambda _1^-(w)[\eta (w)]_-\Big )[\eta (w)]_+\lambda ^+_1(w), \end{aligned}$$

where we put in brackets the terms which need to be normal ordered. Let us focus on the part containing the $\mathsf{a}$s. They give four contributions, which sum up to

10.20 $$\begin{aligned} \mathrm{e}^{-\sum _{m>0}\left( \frac{w}{z}\right) ^m\frac{(1-q^m)(1-t^{-m})}{m(1-q'^m)(1-p^{2m})}\left( 1-2p^m+p^{2m}+\frac{(1-p^{-m})^2p^{4m}}{(1-p^{2m})}\right) }. \end{aligned}$$

The second tensor component arises from

10.21 $$\begin{aligned} \lambda _2^-(z)\lambda _2^+(z)\lambda _2^-(w)\lambda _2^+(w)=\lambda _2^-(z)\Big (\lambda _2^+(z)\lambda _2^-(w)\Big )\lambda _2^+(w), \end{aligned}$$

and the normal ordering function from the part containing the $\mathsf{a}$s reads

10.22 $$\begin{aligned} \mathrm{e}^{-\sum _{m>0}\left( \frac{w}{z}\right) ^m\frac{(1-q^m)(1-t^{-m})}{m(1-q'^m)(1-p^{2m})}\frac{(1-p^{-m})^2p^{3m}}{(1-p^{2m})}}. \end{aligned}$$

Analogous results come from the parts containing the $\mathsf{b}$s but with the replacements $\frac{w}{z}\rightarrow \frac{q' z}{w}, (q,t,p)\rightarrow (q^{-1},t^{-1},p^{-1})$. Multiplying all these contributions together yields

10.23 $$\begin{aligned} \mathrm{e}^{-\sum _{m>0}\left( \frac{w}{z}\right) ^m\frac{(1-q^m)(1-t^{-m})(1-p^m)}{m(1-q'^m)(1-p^{2m})}}\mathrm{e}^{\sum _{m>0}\left( \frac{p^2q' z}{w}\right) ^m\frac{(1-q^{-m})(1-t^{m})(1-p^{-m})}{m(1-q'^m)(1-p^{2m})}}=f_{+,+}(w z^{-1})^{-1}, \end{aligned}$$

where $f_{+,+}(x)$ is defined in (2.11).

## F The refined periodic strip

The amplitude of the periodic strip geometry depicted in Fig. 2 (left) reads as^17^

11.1 $$\begin{aligned}&\mathcal {K}^{\vec \alpha }_{\vec \beta }(Q_m, Q_f;q,t)\!=\!\sum _{\vec \mu ,\vec \nu }\prod _{a=1}^{N}(-Q_{m,a})^{|\mu _a|}\!\prod _{b=1}^{N}(-Q_{f,b})^{|\nu _{b+1}|}\nonumber \\&\qquad \times \prod _{a=1}^N C_{\nu _a\mu _a\alpha _a}(t,q)\!\prod _{b=1}^N C_{\nu _{b+1}^\vee \mu _b^\vee \beta _b^\vee }(q,t)\!\nonumber \\&\quad =\prod _{a=1}^N q^{\frac{\Vert \alpha _a\Vert ^2}{2}}t^{\frac{\Vert \beta ^\vee _a\Vert ^2}{2}}{\tilde{Z}}_{\alpha _a}(t,q){\tilde{Z}}_{\beta _a^\vee }(q,t)\sum _{\vec \mu ,\vec \nu ,\vec \eta ,\vec \sigma }\prod _{a=1}^{N}(-Q_{m,a})^{|\mu _a|}\prod _{b=1}^{N}(-Q_{f,b})^{|\nu _{b+1}|}\nonumber \\&\qquad \times \prod _{a=1}^N s_{\nu ^\vee _a/\eta _a}(p^{-\frac{1}{2}}t^{-\rho }q^{-\alpha _a})s_{\mu _a/\eta _a}(q^{-\rho }t^{-\alpha ^\vee _a})\nonumber \\&\qquad \times \prod _{b=1}^N s_{\nu _{b+1}/\sigma _b}(p^{\frac{1}{2}}t q^{-\rho }t^{-\beta ^\vee _b})s_{\mu ^\vee _b/\sigma _b}(t^{-\rho }q^{-\beta _b}), \end{aligned}$$

where we introduced $p=qt^{-1}$, and

11.2 $$\begin{aligned} {\tilde{Z}}_{\mu }(t,q)=\prod _{(i,j)\in \mu }\frac{1}{1-q^{\mu _i-j}t^{\mu _j^\vee -i+1}}. \end{aligned}$$

We need to evaluate

11.3 $$\begin{aligned} G(x, y,Q_m, Q_f)=\sum _{\vec X,\vec Y}\prod _{a=1}^{2N}(-{\tilde{Q}}_a)^{|X_a|}\prod _{a=1}^{2N}s_{X_a/Y_a}(x_a)s_{X^\vee _a/Y_{a+1}}(y_a), \end{aligned}$$

where we grouped the relevant variables according to

11.4 $$\begin{aligned} X_a&=\left\{ \begin{array}{ll}\mu _\frac{a+1}{2}, &{}\quad a\text { odd }\\ \nu _{\frac{a}{2}+1},&{}\quad a\text { even }\end{array}\right. ,\quad Y_a=\left\{ \begin{array}{ll}\eta _\frac{a+1}{2}, &{}\quad a\text { odd }\\ \sigma _\frac{a}{2},&{}\quad a\text { even }\end{array}\right. \!, \end{aligned}$$

11.5 $$\begin{aligned} {\tilde{Q}}_a&=\left\{ \begin{array}{ll}Q_{m,\frac{a+1}{2}},&{}\quad a\text { odd }\\ Q_{f,\frac{a}{2}},&{}\quad a\text { even }\end{array}\right. \!,\end{aligned}$$

11.6 $$\begin{aligned} (x_a,y_a)&=\left\{ \begin{array}{ll}\left( q^{-\rho }t^{-\alpha ^\vee _\frac{a+1}{2}},t^{-\rho }q^{-\beta _\frac{a+1}{2}}\right) ,&{}\quad a \text { odd }\\ \left( q^{-\rho +\frac{1}{2}}t^{-\beta ^\vee _\frac{a}{2}-\frac{1}{2}},t^{-\rho +\frac{1}{2}}q^{-\alpha _{\frac{a}{2}+1}-\frac{1}{2}}\right) \!,&{}\quad a\text { even }\end{array}\right. \!. \end{aligned}$$

Using standard identities of Schur polynomials [133] we get^18^

11.7 $$\begin{aligned} G(x,y, Q_m, Q_f)=\frac{1}{(q';q')_\infty }\prod _{i,j,k=1}^\infty \prod _{a=1}^{2N}\prod _{\ell =1}^N\frac{1-q'^{k-1}\prod _{s=a}^{a+2\ell -2}{\tilde{Q}}_s~x_{a,i}y_{a+2\ell -2,j}}{1-q'^{k-1}\prod _{s=a}^{a+2\ell -1}{\tilde{Q}}_s~x_{a,i}y_{a+2\ell -1,j}}, \end{aligned}$$

where $q'=\prod _{a=1}^{2N}{\tilde{Q}}_a=\prod _{a=1}^{N}Q_{m,a}Q_{f,a}$, and hence

11.8 $$\begin{aligned}&\mathcal {K}^{\vec \alpha }_{\vec \beta }(Q_m, Q_f;q,t)=\frac{1}{(q';q')_\infty }\prod _{a=1}^N q^{\frac{\Vert \alpha _a\Vert ^2}{2}}t^{\frac{\Vert \beta ^\vee _a\Vert ^2}{2}}{\tilde{Z}}_{\alpha _a}(t,q){\tilde{Z}}_{\beta _a^\vee }(q,t)\nonumber \\&\quad \times \prod _{i,j,k=1}^\infty \prod _{r,\ell =1}^N \frac{(1-q'^{k-1}Q_{r,r+\ell }Q_{m,r}^{-1}q^{-\alpha _{r+\ell ,i}+j-\frac{1}{2}}t^{-\beta ^\vee _{r,j}+i-\frac{1}{2}})}{(1-q'^{k-1}Q_{r,r+\ell }Q_{m,r}^{-1}Q_{m,r+\ell }q^{-\beta _{r+\ell ,i}+j}t^{-\beta ^\vee _{r,j}+i-1})}\nonumber \\&\quad \times \frac{(1-q'^{k-1}Q_{r,r+\ell -1}Q_{m,r+\ell -1}q^{-\beta _{r+\ell -1,i}+j-\frac{1}{2}}t^{-\alpha ^\vee _{r,j}+i-\frac{1}{2}})}{(1-q'^{k-1}Q_{r,r+\ell }q^{-\alpha _{r+\ell ,i}+j-1}t^{-\alpha ^\vee _{r,j}+i})}, \end{aligned}$$

where

11.9 $$\begin{aligned} Q_{a,b}=\left\{ \begin{array}{ll}\prod _{k=a}^{b-1}Q_{m,k}Q_{f,k},&{}\quad a<b\\ q' Q_{b,a}^{-1},&{}\quad a>b\\ 1,&{}\quad a=b\\ q',&{}\quad b=a+N\end{array}\right. . \end{aligned}$$

Using the K-theoretic Nekrasov function (we use the notation of [117])

11.10 $$\begin{aligned} N_{\mu \nu }(Q;q,t)=\prod _{(i,j)\in \mu }(1-Qq^{\mu _i-j}t^{\nu _j^\vee -i+1})\prod _{(i,j)\in \nu }(1-Qq^{-\nu _i+j-1}t^{-\mu _j^\vee +i}), \end{aligned}$$

we also have

11.11 $$\begin{aligned} \prod _{i,j=1}^\infty \frac{1-Qq^{-\nu _i+j-1} t^{-\mu _j^\vee +i}}{1-Qq^{j-1}t^{i}}=N_{\mu \nu }(Q;q,t), \end{aligned}$$

and

11.12 $$\begin{aligned}&\frac{\mathcal {K}^{\vec \alpha }_{\vec \beta }(Q_m,Q_f;q,t)}{\mathcal {K}^{\vec \emptyset }_{\vec \emptyset }(Q_m,Q_f;q,t)} =\prod _{a=1}^N q^{\frac{\Vert \alpha _a\Vert ^2}{2}}t^{\frac{\Vert \beta ^\vee _a\Vert ^2}{2}}{\tilde{Z}}_{\alpha _a}(t,q){\tilde{Z}}_{\beta _a^\vee }(q,t)\nonumber \\&\qquad \times \prod _{r,\ell =1}^N\prod _{k=0}^\infty \frac{N_{\beta _r\alpha _{r+\ell }}\left( p^{\frac{1}{2}} q'^{k}Q_{r,r+\ell }Q_{m,r}^{-1};q,t\right) N_{\alpha _r\beta _{r+\ell -1}}\left( p^{\frac{1}{2}} q'^{k}Q_{r,r+\ell -1}Q_{m,r+\ell -1};q,t\right) }{N_{\beta _r\beta _{r+\ell }}\left( p q'^{k}Q_{r,r+\ell }Q_{m,r}^{-1}Q_{m,r+\ell };q,t\right) N_{\alpha _r\alpha _{r+\ell }}(q'^{k}Q_{r,r+\ell };q,t)}\nonumber \\&\quad =\prod _{a=1}^N q^{\frac{\Vert \alpha _a\Vert ^2}{2}}t^{\frac{\Vert \beta ^\vee _a\Vert ^2}{2}}{\tilde{Z}}_{\alpha _a}(t,q){\tilde{Z}}_{\beta _a^\vee }(q,t)\prod _{k=0}^\infty \frac{N_{\beta _a\alpha _{a}}\left( p^{\frac{1}{2}} q'^{k+1}Q_{m,a}^{-1};q,t\right) N_{\alpha _a\beta _{a}}\left( p^{\frac{1}{2}}q'^{k}Q_{m,b};q,t\right) }{N_{\beta _a\beta _{a}}\left( p q'^{k+1};q,t\right) N_{\alpha _a\alpha _{a}}(q'^{k+1};q,t)}\nonumber \\&\qquad \times \prod _{1\le a\ne b\le N}\prod _{k=0}^\infty \frac{N_{\beta _a\alpha _{b}}\left( p^{\frac{1}{2}} q'^{k}Q_{ab}Q_{m,a}^{-1};q,t\right) N_{\alpha _a\beta _{b}}\left( p^{\frac{1}{2}} q'^{k}Q_{a,b}Q_{m,b};q,t\right) }{N_{\beta _a\beta _{b}}\left( p q'^{k}Q_{a,b}Q_{m,a}^{-1}Q_{m,b};q,t\right) N_{\alpha _a\alpha _{b}}(q'^{k}Q_{ab};q,t)}, \end{aligned}$$

where we used $q'=Q_{a,b}Q_{b,a}$ for $a\ne b$ and $Q_{a,a+N}=q'$. Introducing the elliptic version of the Nekrasov function^19^ ^20^

11.13 $$\begin{aligned} N_{\mu \nu }(Q;q,t|q')&=\prod _{(i,j)\in \mu }\Theta (Qq^{\mu _i-j}t^{\nu _j^\vee -i+1};q')\prod _{(i,j)\in \nu }\Theta (Qq^{-\nu _i+j-1}t^{-\mu _j^\vee +i};q'), \end{aligned}$$

11.14 $$\begin{aligned}&=\prod _{k=0}^\infty N_{\mu \nu }(q'^kQ;q,t)N_{\nu \mu }(pq'^{k+1}Q^{-1};q,t), \end{aligned}$$

we also have

11.15 $$\begin{aligned}&\frac{\mathcal {K}^{\vec \alpha }_{\vec \beta }( Q_m, Q_f;q,t)}{\mathcal {K}^{\vec \emptyset }_{\vec \emptyset }(Q_m, Q_f;q,t)} =\prod _{a=1}^N \frac{q^{\frac{\Vert \alpha _a\Vert ^2}{2}}t^{\frac{\Vert \beta ^\vee _a\Vert ^2}{2}}{\tilde{Z}}_{\alpha _a}(t,q){\tilde{Z}}_{\beta _a^\vee }(q,t)}{N_{\beta _a\beta _{a}}(p q'^{k+1};q,t)N_{\alpha _a\alpha _{a}}(q'^{k+1};q,t)}\nonumber \\&\quad \times \frac{\prod _{a,b=1}^N N_{\alpha _{a}\beta _{b}}(p^{\frac{1}{2}} Q_{a,b}Q_{m,b};q,t|q')}{\prod _{1\le a\ne b\le N}\prod _{k=0}^\infty N_{\beta _a\beta _{b}}(p q'^{k}Q_{a,b}Q_{m,a}^{-1}Q_{f,a};q,t)N_{\alpha _a\alpha _{b}}(q'^{k}Q_{a,b};q,t)}.\nonumber \\ \end{aligned}$$

The denominator cannot be expressed solely in terms of the elliptic Nekrasov functions because of the “wrong” *q*, *t* shift. This phenomenon has been related to the anomalous modular transformation property of the open periodic strip [108].

The $\mathbb {R}^4\times \mathbb {T}^2$ Nekrasov instanton partition function of the *U*(*N*) theory with *N* fundamental and anti-fundamental hypers can be computed by gluing two periodic strips, with the result

11.16 $$\begin{aligned} \mathcal {Z}_{\mathrm{inst}}^{\mathbb {R}^4\times \mathbb {T}^2}&=\sum _{\vec Y}\prod _{a=1}^N(-Q_{B,a})^{|Y^a|}\frac{\mathcal {K}^{\vec Y}_{\vec \emptyset }( Q_m, Q_f;q,t)}{\mathcal {K}^{\vec \emptyset }_{\vec \emptyset }(Q_m,Q_f;q,t)}\frac{\mathcal {K}^{\vec \emptyset }_{\vec Y}({{\bar{Q}}}_m,{{\bar{Q}}}_f;q,t)}{\mathcal {K}^{\vec \emptyset }_{\vec \emptyset }({{\bar{Q}}}_m,{{\bar{Q}}}_f;q,t)}\nonumber \\&=\sum _{\vec Y}\prod _{a=1}^N(-Q_{B,a})^{|Y^a|}\prod _{a=1}^N\frac{q^{\frac{\Vert Y^a\Vert ^2}{2}}t^{\frac{\Vert {Y^a}^\vee \Vert ^2}{2}}{\tilde{Z}}_{Y^a}(t,q){\tilde{Z}}_{{Y^a}^\vee }(q,t)}{\prod _{k=0}^\infty N_{Y^aY^a}(p q'^{k+1};q,t)N_{Y^aY^a}(q'^{k+1};q,t)}\nonumber \\&~~~\times \frac{\prod _{a,b=1}^N N_{\emptyset Y^b}(p^{\frac{1}{2}}Q_{ab}{\bar{Q}}_{m,a};q,t|q') N_{Y^a\emptyset }(p^{\frac{1}{2}}Q_{ab}Q_{m,b};q,t|q')}{\prod _{1\le a\ne b \le N} N_{Y^a Y^b}(Q_{ab};q,t|q')}. \end{aligned}$$

Using

11.17 $$\begin{aligned} q^{\frac{\Vert \mu \Vert ^2}{2}}t^{\frac{\Vert \mu ^\vee \Vert ^2}{2}}{\tilde{Z}}_\mu (t,q){\tilde{Z}}_{\mu ^\vee }(q,t)=\frac{(-p^{-\frac{1}{2}})^{|\mu |}}{N_{\mu \mu }(1;q,t)}, \end{aligned}$$

we can recast the second line of (11.16) as

11.18 $$\begin{aligned} \frac{\prod _{a=1}^N (p^{-\frac{1}{2}}Q_{B,a})^{| Y^a|}}{\prod _{a=1}^N N_{Y^aY^a}(1;q,t|q')}, \end{aligned}$$

and hence

11.19 $$\begin{aligned} \mathcal {Z}_{\mathrm{inst}}^{\mathbb {R}^4\times \mathbb {T}^2}= & {} \sum _{\vec Y}\prod _{a=1}^N (p^{-\frac{1}{2}}Q_{B,a})^{|Y^a|}\nonumber \\&\times \prod _{a,b=1}^N\frac{N_{\emptyset Y^b}(p^{\frac{1}{2}}Q_{ab}{\bar{Q}}_{m,a};q,t|q') N_{Y^a\emptyset }(p^{\frac{1}{2}} Q_{ab}Q_{m,b};q,t|q')}{N_{Y^a Y^b}(Q_{ab};q,t|q')}.\nonumber \\ \end{aligned}$$

In order to make contact with the field theory language, it is useful to introduce the parametrization

11.20 $$\begin{aligned} Q_{ab}=\left\{ \begin{array}{ll}A_a A_b^{-1},&{}\quad a\le b\\ q' A_aA_b^{-1},&{}\quad a>b\end{array}\right. ,\quad Q_{m,a}=A_aQ_a p^{-\frac{1}{2}},\quad {\bar{Q}}_{m,a}=A_a^{-1} {\bar{Q}}_a p^{-\frac{1}{2}}, \end{aligned}$$

so that (4.5) is automatically satisfied. Using the shift property

11.21 $$\begin{aligned} N_{\mu \nu }(q' Q|q')\!=\!N_{\mu \nu }(Q|q')~(-Q)^{-|\mu |-|\nu |}q^{\frac{1}{2}(|\nu |+\Vert \nu \Vert ^2+|\mu |\!-\!\Vert \mu \Vert ^2)}t^{-\frac{1}{2}(|\mu |\!-\!\Vert \mu ^\vee \Vert ^2\!+\!|\nu |\!+\!\Vert \nu ^\vee \Vert ^2)}, \end{aligned}$$

we can rewrite

11.22 $$\begin{aligned} \mathcal {Z}_{\mathrm{inst}}^{\mathbb {R}^4\times \mathbb {T}^2}= & {} \sum _{\vec Y}\prod _{a=1}^N\left( \frac{p^{-\frac{1}{2}}Q_{B,a}}{\prod _{b=1}^N\left( p^\frac{1}{2}{\bar{Q}}_{m,b}\right) }\right) ^{|Y^a|} \frac{\prod _{b\le a}\left( p^\frac{1}{2}{\bar{Q}}_{m,b}\right) ^{|Y^a|}}{\prod _{b<a}\left( p^\frac{1}{2}Q_{m,b}\right) ^{|Y^a|}}\nonumber \\&\times \prod _{a,b=1}^N\frac{N_{\emptyset Y^b}(A_b^{-1}{\bar{Q}}_a;q,t|q') N_{Y^a\emptyset }(A_a Q_b;q,t|q')}{N_{Y^a Y^b}(A_a A_b^{-1};q,t|q')}. \end{aligned}$$

Generically, for *M* glued strips labeled by (*c*) we have [134]

11.23 $$\begin{aligned} \prod _a {Q^{(c)}_{B,a}}^{|Y_a^{(c)}|}=\left( Q_{B,1}^{(c)}p^\frac{1}{2}Q_{m,1}^{(c-1)}\right) ^{\sum _a|Y^{(c)}_a|}\frac{\prod _{b<a}\left( p^\frac{1}{2}Q^{(c)}_{m,b}\right) ^{|Y^{(c)}_a|}}{\prod _{b \le a}\left( p^\frac{1}{2} Q^{(c-1)}_{m,b}\right) ^{|Y^{(c)}_a|}}, \end{aligned}$$

so that we can define a renormalized expansion parameter ${\tilde{Q}}_B$ and write (4.6).

## Footnotes

1 This is a function invariant w.r.t. to *q*-shifts of its arguments, i.e., $c_\beta (z;q)|_{z_i\rightarrow q z_i}=c_\beta (z;q)$.

2 We identify $$\alpha _n^\dagger =\alpha _{-n}, \beta _n^\dagger =\beta _{-n}$$.

3 For the role of Dotsenko–Fateev integrals in the 5d AGT duality we refer to [25, 26, 28, 29, 47, 56].

4 In order to avoid cluttering, we will denote $\frac{\mathrm{d}z}{2\pi \mathrm{i}z}\!\!=\!\!\frac{\prod _j \mathrm{d}z_j}{\prod _j 2\pi \mathrm{i}z_j}$, with the range of *j* clear from the context.

5 We will see that this is also related to the discussion around (2.22).

6 The 6d origin of *t* will be clarified in Sect. 4.

7 See also an analogous discussion in appendix C of [25].

8 A relation between $\mathcal {N}=2$ theories on $S^2\!\times \!\mathbb {T}^2$ and M-strings (see below) is discussed in [105].

9 For a mathematical definition of the instanton partition function we refer to [4, 5, 18, 19, 116].

10 This relation was anticipated in [118], where a review of factorization of supersymmetric partition functions in various dimensions and for diverse compact spaces, 4d and 5d AGT can also be found.

11 See also [123] for another elliptic deformation, and [126] for its application to 3d/5d coupled supersymmetric gauge theories and integrable models.

12 Some of these aspects have been considered in the related work [129].
